# Proximate and Ultimate Perspectives on Romantic Love

**DOI:** 10.3389/fpsyg.2021.573123

**Published:** 2021-04-12

**Authors:** Adam Bode, Geoff Kushnick

**Affiliations:** Human Behavioural Ecology Research Group, School of Archaeology and Anthropology, ANU College of Arts and Social Sciences, The Australian National University, Canberra, ACT, Australia

**Keywords:** romantic love, mechanisms, ontogeny, functions, phylogeny, Tinbergen, human mating, definition

## Abstract

Romantic love is a phenomenon of immense interest to the general public as well as to scholars in several disciplines. It is known to be present in almost all human societies and has been studied from a number of perspectives. In this integrative review, we bring together what is known about romantic love using Tinbergen’s “four questions” framework originating from evolutionary biology. Under the first question, related to mechanisms, we show that it is caused by social, psychological mate choice, genetic, neural, and endocrine mechanisms. The mechanisms regulating psychopathology, cognitive biases, and animal models provide further insights into the mechanisms that regulate romantic love. Under the second question, related to development, we show that romantic love exists across the human lifespan in both sexes. We summarize what is known about its development and the internal and external factors that influence it. We consider cross-cultural perspectives and raise the issue of evolutionary mismatch. Under the third question, related to function, we discuss the fitness-relevant benefits and costs of romantic love with reference to mate choice, courtship, sex, and pair-bonding. We outline three possible selective pressures and contend that romantic love is a suite of adaptions and by-products. Under the fourth question, related to phylogeny, we summarize theories of romantic love’s evolutionary history and show that romantic love probably evolved in concert with pair-bonds in our recent ancestors. We describe the mammalian antecedents to romantic love and the contribution of genes and culture to the expression of modern romantic love. We advance four potential scenarios for the evolution of romantic love. We conclude by summarizing what Tinbergen’s four questions tell us, highlighting outstanding questions as avenues of potential future research, and suggesting a novel ethologically informed working definition to accommodate the multi-faceted understanding of romantic love advanced in this review.

## Introduction

Romantic love is a complex suite of adaptations and by-products that serves a range of functions related to reproduction (Fletcher et al., 2015; Buss, 2019). It often occurs early in a romantic relationship but can lead to long-term mating. It is a universal or near-universal (Jankowiak and Fischer, 1992; Gottschall and Nordlund, 2006; Jankowiak and Paladino, 2008; Fletcher et al., 2015; Buss, 2019; Sorokowski et al., 2020) and is characterized by a range of cognitive, emotional, behavioral, social, genetic, neural, and endocrine activity. It occurs across the lifespan in both sexes. Romantic love serves a variety of functions that vary according to life-stage and duration, including mate choice, courtship, sex, and pair-bonding. Its evolutionary history is probably coupled with the emergence of pair-bonds relatively recently in human evolutionary history.

Romantic love has received attention from scholars in diverse fields, including neurobiology, endocrinology, psychology, and anthropology. Our review aims to synthesize multiple threads of knowledge into a more well-rounded perspective on romantic love. To accomplish this, we do the following: First, we lay out our analytical framework based on Tinbergen’s (1963) “four questions” for explaining a biological phenomenon. Second, using this framework as an organizing tool, we summarize what is known about the social mechanisms, psychological mate choice mechanisms, genetics, neurobiology, endocrinology, development across the lifetime of an individual, fitness-relevant functions, and evolutionary history of romantic love. Finally, we conclude by summarizing what Tinbergen’s four questions tell us, identifying areas for future research, and providing a new ethologically informed working definition of romantic love.

### Analytical Framework

Much work has been done to examine romantic love as a biological characteristic. Numerous reviews have described the neurobiology and endocrinology of romantic love (e.g., Fisher, 2004, 2006; Zeki, 2007; Hatfield and Rapson, 2009; Reynaud et al., 2010; Cacioppo et al., 2012b; de Boer et al., 2012; Diamond and Dickenson, 2012; Dunbar, 2012; Tarlaci, 2012; Xu et al., 2015; Fisher et al., 2016; Zou et al., 2016; Tomlinson et al., 2018; Walum and Young, 2018; Cacioppo, 2019). Two meta-analyses (Ortigue et al., 2010; Cacioppo et al., 2012a) considered fMRI studies of romantic love. There have been some accounts of romantic love or love from an evolutionary perspective (e.g., Hendrick and Hendrick, 1991; Fisher, 1995, 2016; Fisher et al., 2006, 2016; Kenrick, 2006; Lieberman and Hatfield, 2006; Schmitt, 2006; Fletcher et al., 2015; Sorokowski et al., 2017; Buss, 2019).

No one, however, has addressed the full spectrum of approaches used in biology to provide a comprehensive account of romantic love. We fill this gap by framing our review of romantic love around Tinbergen’s (1963) “four questions” for explaining biological traits. It was developed in the context of trying to provide a holistic, integrative understanding of animal behavior, and is an extension of earlier explanatory frameworks, including Mayr’s (1961) distinction between proximate and ultimate explanations in biology (Bateson and Laland, 2013). It includes two proximate explanations, mechanistic and ontogenetic, and two ultimate (evolutionary) explanations, functional and phylogenetic. To illustrate the use of this framework, we refer to elements of Zeifman’s (2001) analysis of infant crying as a biological trait using this framework. An outline of our use of this framework is presented in Table 1.

**TABLE 1:** Summary of romantic love using Tinbergen’s (1963) framework.

**Causes**	**Key question**	**Summary of answer**
**Proximate**		
Mechanisms	What are the mechanisms that cause romantic love?	Romantic love is associated with social mechanisms, psychological mate choice mechanisms, and the expression of specific genes. The cognitive, emotional, and behavioral features of romantic love result from neural activity associated with reward and motivation, emotions, sexual desire and arousal, and social cognition as well as endocrine activity associated with sex hormones, serotonin, dopamine, oxytocin, cortisol, and nerve growth factor. Research into psychopathology, cognitive biases, and animal models can inform our understanding of the mechanisms regulating romantic love.
Ontogeny	How does romantic love develop over the lifetime of an individual?	Romantic love first develops in childhood, manifests at all ages, usually lasts months or years, but can exist for many years or decades. It is influenced by a range of internal and external factors, is cross-cultural, and may be influenced by the modern environment.
**Ultimate**		
Functions	What are the fitness-relevant functions of romantic love?	Romantic love has a number of fitness-relevant benefits and costs that relate to four interrelated functions: mate choice, courtship, sex, and pair-bonding. There is a small amount of evidence about the health benefits and costs of romantic love. Theories exist about selective pressures that led to the evolution of romantic love. Romantic love is a complex suite of adaptations and by-products and can be either adaptive or maladaptive.
Phylogeny	What is the evolutionary history of romantic love?	The antecedents to romantic love existed in mammals before romantic love evolved. Its evolutionary history was probably coupled with the emergence of pair-bonds sometime recently in human evolution. There may be ethnic or geographic variation in romantic love and culture has influenced romantic love’s effect on human evolution in recent times.

Proximate explanations focus on the workings of biological and social systems and their components, both on a short-term (mechanistic) and longer-term (ontogenetic) basis (Tinbergen, 1963; Zeifman, 2001). Mechanistic explanations attempt to answer questions about how behavior is produced by an organism. It is about the immediate causation of the behavior. A baby’s cry, under this class of explanation, might be viewed as an expression of emotion regulated by the limbic system. In our analysis, we ask: “What are the mechanisms that cause romantic love?” Ontogenetic explanations attempt to answer questions about how the behavior develops over the life course. A baby’s cry, thus, might be viewed as a vocalization that changes in frequency and context over the first year of life, and then across the rest of childhood. In our analysis, we ask: “How does romantic love develop over the lifetime of an individual?”

Ultimate explanations focus on the application of evolutionary logic to understand behavior, both on a short-term (functional) and long-term (phylogenetic) basis (Tinbergen, 1963; Zeifman, 2001). Functional explanations attempt to answer questions about the fitness consequences of behavior and how it functions as an adaptation. A baby’s cry, thus, might be viewed as an adaptation that enhances offspring survival by eliciting care or providing information about its state. As the fitness consequences may be negative as well, it might focus on both benefits and costs. For instance, the cry may decrease survival by attracting predators or depleting scarce energy reserves. In our analysis, we ask: “What are the fitness-relevant functions of romantic love?” Phylogenetic explanations attempt to answer questions about the evolutionary history of a behavior and the mechanisms that produce it. A baby’s cry, thus, might be understood from the perspective of whether similar behaviors are present in closely related species. In our analysis, we ask: “What is the evolutionary history of romantic love?”

Tinbergen’s (1963) framework has been a useful tool for organizing research and theory on behavior and other biological traits across all major kingdoms of life, from plants (e.g., Satake, 2018) to humans (e.g., Winterhalder and Smith, 1992; Zeifman, 2001; Stephen et al., 2017; Luoto et al., 2019). It allows us to build holistic explanations of biological phenomena by examining complementary, but often non-mutually exclusive, categories of explanation (Bateson and Laland, 2013). We believe that this approach to understanding romantic love will clarify the usefulness and interdependence of the various aspects of the biology of romantic love without falling into the pitfalls of posing explanations for the phenomena that are in opposition rather than complementary (Nesse, 2013).

### Definitions

There are a number of definitions and descriptions of romantic love. These definitions and descriptions have different names for romantic love, but all are attempting to define the same construct. We present, here, four definitions or descriptions of romantic love that continue to have relevance to contemporary research.

Walster and Walster (1978) were among the first to scientifically define romantic love. They gave it the name “passionate love” and their definition has been revised several times (e.g., Hatfield and Walster, 1985; Hatfield and Rapson, 1993). A definition of passionate love is:

A state of intense longing for union with another. Passionate love is a complex functional whole including appraisals or appreciations, subjective feelings, expressions, patterned physiological processes, action tendencies, and instrumental behaviors. Reciprocated love (union with the other) is associated with fulfillment and ecstasy; unrequited love (separation) with emptiness, anxiety, or despair (Hatfield and Rapson, 1993, p. 5).

Hendrick and Hendrick (1986) propose a description of romantic love in the context of describing six different “love styles” (Lee, 1976). They label it “eros.” It too has undergone some changes. A recent version of the description is:

Strong physical attraction, emotional intensity, a preferred physical appearance, and a sense of inevitability of the relationship define the central core of eros. Eros can “strike” suddenly in a revolution of feeling and thinking (Hendrick and Hendrick, 2019, p. 244).

Sternberg (1986) provides a description of romantic love based on three components of love in close relationships: intimacy, passion and commitment. He calls it “romantic love” and describes it as such:

This kind of love derives from a combination of the intimacy and passion components of love. In essence, it is liking with an added element, namely, the arousal brought about by physical attraction and its concomitants. According to this view, then, romantic lovers are not only drawn physically to each other but are also bonded emotionally (Sternberg, 1986, p. 124).

A more recent definition of romantic love informed by evolutionary theory has been proposed by Fletcher et al. (2015). Rather than providing a discrete series of sentences, they propose a working definition of “romantic love” that is explained with reference to some of the psychological research on romantic love and by summarizing five distinct features of romantic love. These features are:

(1) Romantic love is a powerful commitment device, composed of passion, intimacy, and caregiving;
(2) Romantic love is universal and is associated with pair-bonding across cultures;
(3) Romantic love automatically suppresses effort and attention given to alternative partners;
(4) Romantic love has distinct emotional, behavioral, hormonal, and neuropsychological features; and
(5) Successful pair-bonding predicts better health and survival across cultures for both adults and offspring (Fletcher et al., 2015, p. 22).

Despite these attempts to define and describe romantic love, no single term or definition has been universally adopted in the literature. The psychological literature often uses the terms “romantic love,” “love,” and “passionate love” (e.g., Sternberg and Sternberg, 2019). Seminal work called it “limerence” (Tennov, 1979). The biological literature generally uses the term “romantic love” and has investigated “early stage intense romantic love” (e.g., Xu et al., 2011), “long-term intense romantic love” (e.g., Acevedo et al., 2012), or being “in love” (e.g., Marazziti and Canale, 2004). In this review, what we term “romantic love” encompasses all of these definitions, descriptions, and terms. Romantic love contrasts with “companionate love,” which is felt less intensely, often follows a period of romantic love (Hatfield and Walster, 1985), and merges feelings of intimacy and commitment (Sternberg, 1986).

#### Psychological Characteristics

Hatfield and Sprecher (1986) theoretically developed the Passionate Love Scale to assess the cognitive, emotional, and behavioral components of romantic love among people who are in a relationship. There are other ways of measuring romantic love (Hatfield et al., 2012), and some, such as Sternberg’s Triangular Love Scale (Sternberg, 1997; Sumter et al., 2013) or the Love Attitudes Scale (Hendrick and Hendrick, 1986; Hendrick et al., 1998), measure the same constructs (Masuda, 2003; Graham, 2011). The Passionate Love Scale is only valid in people who are in a romantic relationship with their loved one. Regardless, the Passionate Love Scale provides a particularly useful account of some of the psychological characteristics of romantic love. It has been used widely in research investigating romantic love in relationships (Feybesse and Hatfield, 2019).

Cognitive components of romantic love include intrusive thinking or preoccupation with the partner, idealization of the other in the relationship, and desire to know the other and to be known. Emotional components include attraction to the other, especially sexual attraction, negative feelings when things go awry, longing for reciprocity, desire for complete union, and physiological arousal. Behavioral components include actions toward determining the other’s feelings, studying the other person, service to the other, and maintaining physical closeness (Hatfield and Sprecher, 1986).

Romantic love shares a number of physiological and psychological characteristics with addiction. “[T]hey focus on their beloved (salience); and they yearn for their beloved (craving). They feel a “rush” of exhilaration when seeing or thinking about him or her (euphoria/intoxication). As their relationship builds, the lover experiences the common signs of drug withdrawal, too, including protest, crying spells, lethargy, anxiety, insomnia, or hypersomnia, loss of appetite or binge eating, irritability and chronic loneliness.” (Fisher et al., 2016, p. 2) A number of reviews have highlighted the behavioral and neurobiological similarities between addiction and romantic love (e.g., Reynaud et al., 2010; Fisher et al., 2016; Zou et al., 2016).

There is evidence that romantic love is associated with increased hypomanic symptoms (elevated mood, Brand et al., 2007; Bajoghli et al., 2011, 2013, 2014, 2017; Brand et al., 2015), a change (increase or decrease) in depression symptoms (Stoessel et al., 2011; Bajoghli et al., 2013, 2014, 2017; Price et al., 2016; Verhallen et al., 2019; Kuula et al., 2020), and increased state anxiety (Hatfield et al., 1989; Wang and Nguyen, 1995; Bajoghli et al., 2013, 2014, 2017; Brand et al., 2015; Kuula et al., 2020). See Supplementary Table 1 for information about studies investigating hypomania, depression, and anxiety symptoms in people experiencing romantic love. Romantic love is also characterized by cognitive biases which resemble “positive illusions,” which are a tendency to perceive one’s relationship and one’s loved one in a positive light or bias (Song et al., 2019).

## Proximate Perspectives

### Mechanisms

When applied to romantic love, the first of Tinbergen’s (1963) four questions asks: “What are the mechanisms that cause romantic love?” This can be answered with reference to social mechanisms, psychological mate choice mechanisms, genetics, neurobiology, and endocrinology (Zeifman, 2001; Bateson and Laland, 2013). Research into the social mechanisms and genetics of romantic love are in their infancy, but there is substantial theory on psychological mate choice mechanisms and ample research has been undertaken into the neural and endocrine activity associated with romantic love. Additional insights can be garnered from the neurobiology and endocrinology of psychopathology, cognitive biases, and animal models.

#### Social Mechanisms

Some precursors to romantic love (others discussed below) that act strongly as social mechanisms that cause romantic love are reciprocal liking, propinquity, social influence, and the filling of needs (e.g., Aron et al., 1989; Pines, 2001; Riela et al., 2010). Reciprocal liking (mutual attraction) is “being liked by the other, both in general, as well as when it is expressed through self-disclosure” (Aron et al., 1989, p. 245). It has been frequently identified as preceding romantic love among participants from the United States and is cross-culturally identified as the strongest preference in mates among both sexes (Buss et al., 1990). “Whether expressed in a warm smile or a prolonged gaze, the message is unmistakable: ‘It’s safe to approach, I like you too. I’ll be nice. You’re not in danger of being rejected”’ (Hazan and Diamond, 2000, p. 197). Reciprocal liking may encourage the social approach and courtship activities characteristic and causative of romantic love.

Propinquity is “familiarity, in terms of having spent time together, living near the other, mere exposure to the other, thinking about the other, or anticipating interaction with the other” (Aron et al., 1989, p. 245). It has more recently been named “familiarity” (see Riela et al., 2010). The extended exposure of an individual to another helps to cause romantic love and specifically facilities the development of romantic love over extended periods of time. Propinquity, in our evolutionary history, served to ensure that “potential mates who are encountered daily at the river’s edge have an advantage over those residing on the other side” (Hazan and Diamond, 2000, p. 201). Given that the pool of potential mates in our evolutionary history would have been limited by the size of the groups in which we lived and the fact that most individuals of reproductive age would already have been involved in long-term mating relationships, propinquity is likely to have played a particularly important role in the generation of romantic love. Until recently (to a somewhat lesser extent, today), with the wide-scale take-up of online dating, propinquity played a role in the formation of many long-term pair-bonds, and presumably, romantic love, as is evidenced by a relatively high proportion of people having met their romantic partners in the places where exposure was facilitated, such as school, college, or work (Rosenfeld et al., 2019). Changes in the importance of certain precursors in causing romantic love may be the result of a mismatch between the modern environment and our genotypes that evolved in a very different environment (discussed in detail below; see Li et al., 2018).

Social influences are “both general social norms and approval of others in the social network” (Aron et al., 1989, p. 245). This may cause people to fall in love with others who are of a similar attractiveness, cultural group, ethnic group, profession, economic class, or who are members of the same social group. Social influences may, directly, impact who we fall in love with by providing approval to a romantic union or, indirectly, by facilitating propinquity. The effect of social influences is demonstrated in the relatively large number of people who met their romantic partner through friends (Rosenfeld et al., 2019). The filling of needs is “having the self’s needs met or meeting the needs of the other (e.g., he makes me happy, she buys me little presents that show she cares), and typically implies characteristics that are highly valued and beneficial in relationship maintenance (e.g., compassion, respect)” (Riela et al., 2010, pp. 474–475). The filling of needs may cause romantic love when social interaction facilitates a union where both partners complement each other.

#### Psychological Mate Choice Mechanisms

Mate choice, in the fields of evolutionary theory, can be defined as “the process that occurs whenever the effects of traits expressed in one sex lead to non-random allocation of reproductive investment with members of the opposite sex” (Edward, 2015, p. 301). It is essentially the process of intersexual selection proposed by Darwin (2013) more than 150 years ago (Darwin, 1859) whereby someone has a preference for mating with a particular individual because of that individual’s characteristics. Mate choice, to that extent, involves the identification of a desirable conspecific (Fisher et al., 2005) and sometimes, the focusing of mating energies on that individual. Mate preferences, sexual desire, and attraction all contribute to romantic love. The concepts of “extended phenotypes” and “overall attractiveness” help to explain how these features operate. Romantic love, as discussed below, serves a mate choice function (Fisher et al., 2005) and these mechanisms and constructs contribute to when, and with whom, an individual falls in love.

A large body of research has developed around universal mate preferences (e.g., Buss and Barnes, 1986; Buss, 1989; Buss et al., 1990; Buss and Schmitt, 2019; Walter et al., 2020). Women, more than men, show a strong preference for resource potential, social status, a slightly older age, ambition and industriousness, dependability and stability, intelligence, compatibility, certain physical indicators, signs of good health, symmetry, masculinity, love, kindness, and commitment (Buss, 1989, 2016; Walter et al., 2020). Men, more than women, have preferences for youth, physical beauty, certain body shapes, chastity, and fidelity (Buss, 1989, 2016). Both sexes have particularly strong preferences for kindness and intelligence (Buss et al., 1990). A male-taller-than-female norm exists in mate preferences and there is some evidence that women have a preference for taller-than-average height (e.g., Salska et al., 2008; Yancey and Emerson, 2014). Mutual attraction and reciprocated love are the most important characteristics that both women and men look for in a potential partner (Buss et al., 1990).

Mate choice and attraction may be based on assessments of “extended phenotypes” (Dawkins, 1982; Luoto, 2019a), which include biotic and abiotic features of the environment that are influenced by an individual’s genes. For example, an extended phenotype would include an individual’s dwelling, car, pets, and social media presence. These can convey information relevant to fitness. Overall mate attractiveness, which is constituted by signs of health and fertility, neurophysiological efficiency, provisioning ability and resources, and capacity for cooperative relationships (Miller and Todd, 1998) may be another heuristic through which attraction and mate choice operate.

Many mate preferences are relatively universal and therefore are likely to have at least some genetic basis (as suggested by, Sugiyama, 2015). While mate preferences are linked to actual mate selection (Li et al., 2013; Li and Meltzer, 2015; Conroy-Beam and Buss, 2016; Buss and Schmitt, 2019), strong mate preferences do not always translate into real-world mate choice (Todd et al., 2007; Stulp et al., 2013). This is in part because mate preferences function in a tradeoff manner whereby some preferences are given priority over others (see Li et al., 2002; Thomas et al., 2020). That is, mate choice is a multivariate process that includes the integration and tradeoff of several preferences (Conroy-Beam et al., 2016). Mate preferences are important because they may serve as a means of screening potential mates, while sexual desire and attraction operationalize these preferences, and romantic love crystalizes them.

Sexual desire and attraction may be antecedents to falling in love and there is evidence that physiologically, sexual desire progresses into romantic love within shared neural structures (Cacioppo et al., 2012a). However, although both sexual desire and attraction operationalize mate choice, only attraction, and not sexual desire, may be necessary for romantic love to occur (see Leckman and Mayes, 1999; Diamond, 2004). Intense attraction is characterized by increased energy, focused attention, feelings of exhilaration, intrusive thinking, and a craving for emotional union (Fisher, 1998) although it exists on a spectrum of intensity.

#### Genetics

Changes in the expression of at least 61 genes are associated with falling in love in women (Murray et al., 2019) suggesting that these genes may regulate features of romantic love. The DRD2 *Taq*I A polymorphism, which regulates Dopamine 2 receptor density (Jonsson et al., 1999), is associated with eros (Emanuele et al., 2007). Polymorphisms of genes that regulate vasopressin receptors (AVPR1a rs3), oxytocin receptors (OXTR rs53576), dopamine 4 receptors (DRD4-7R), and dopamine transmission (COMT rs4680) are associated with activity in the ventral tegmental area which, in turn, is associated with eros in newlyweds (Acevedo et al., 2020).

#### Neurobiology

Neuroimaging studies (see Supplementary Table 2) implicate dozens of brain regions in romantic love. We focus, here, on only some of the most frequently replicated findings in an attempt to simplify a description of the neural activity associated with romantic love and explain its psychological characteristics. Romantic love, at least in people who are in a relationship with their loved one, appears to be associated with activity (activation or deactivation compared with a control condition) in four main overlapping systems: reward and motivation, emotions, sexual desire and arousal, and social cognition.

Reward and motivation structures associated with romantic love include those found in the mesolimbic pathway: the ventral tegmental area, nucleus accumbens, amygdala, and medial prefrontal cortex (Xu et al., 2015). Activity in the mesolimbic pathway substantiates the claim that romantic love is a motivational state (Fisher et al., 2005) and helps to explain why romantic love is characterized by psychological features such as longing for reciprocity, desire for complete union, service to the other, maintaining physical closeness, and physiological arousal (Hatfield and Sprecher, 1986).

Emotional centers of the brain associated with romantic love include the amygdala, the anterior cingulate cortex (Bartels and Zeki, 2000; Aron et al., 2005; Fisher et al., 2010; Younger et al., 2010; Zeki and Romaya, 2010; Stoessel et al., 2011; Acevedo et al., 2012; Scheele et al., 2013; Song et al., 2015), and the insula (Bartels and Zeki, 2000; Aron et al., 2005; Ortigue et al., 2007; Fisher et al., 2010; Younger et al., 2010; Zeki and Romaya, 2010; Stoessel et al., 2011; Acevedo et al., 2012; Xu et al., 2012b; Song et al., 2015). Activity in these structures helps to explain romantic love’s emotional features such as negative feelings when things go awry, longing for reciprocity, desire for complete union, and physiological arousal (Hatfield and Sprecher, 1986).

The primary areas associated with both romantic love and sexual desire and arousal include the caudate, insula, putamen, and anterior cingulate cortex (Diamond and Dickenson, 2012). The involvement of these regions helps to explain why people experiencing romantic love feel extremely sexually attracted to their loved one (Hatfield and Sprecher, 1986). The neural similarities and overlapping psychological characteristics of romantic love and sexual desire are well documented (see Hatfield and Rapson, 2009; Cacioppo et al., 2012a; Diamond and Dickenson, 2012).

Social cognition centers in the brain repeatedly associated with romantic love include the amygdala, the insula (Adolphs, 2001), and the medial prefrontal cortex (Van Overwalle, 2009). Social cognition plays a role in the social appraisals and cooperation characteristics of romantic love. Activity in these regions helps to explain psychological characteristics such as actions toward determining the other’s feelings, studying the other person, and service to the other (Hatfield and Sprecher, 1986).

In addition to activity in these four systems, romantic love is associated with activity in higher-order cortical brain areas that are involved in attention, memory, mental associations, and self-representation (Cacioppo et al., 2012b). Mate choice (a function of romantic love detailed below) has been specifically associated with the mesolimbic pathway and hypothalamus (Calabrò et al., 2019). The mesolimbic pathway, thalamus, hypothalamus, amygdala, septal region, prefrontal cortex, cingulate cortex, and insula have been specifically associated with human sexual behavior (Calabrò et al., 2019), which has implications for the sex function of romantic love (detailed below).

Isolated studies have identified sex differences in the neurobiological activity associated with romantic love. One study (Bartels and Zeki, 2004) found activity in the region ventral to the genu in only women experiencing romantic love. One preliminary study of romantic love (see Fisher et al., 2006) found that “[m]en tended to show more activity than women in a region of the right posterior dorsal insula that has been correlated with penile turgidity and male viewing of beautiful faces. Men also showed more activity in regions associated with the integration of visual stimuli. Women tended to show more activity than men in regions associated with attention, memory and emotion” (p. 2181).

#### Endocrinology

Romantic love is associated with changes in circulating sex hormones, serotonin, dopamine, oxytocin, cortisol, and nerve growth factor systems. Table 2 presents the endocrine factors which are found to be different, compared to controls, in people experiencing romantic love. More information about the controlled studies discussed in this subsection is presented in Supplementary Table 3. Endocrine factors associated with romantic love have most of their psychological and other effects because of their role as a hormone (e.g., sex hormones, cortisol) or neurotransmitter (e.g., serotonin, dopamine), although many factors operate as both (see Calisi and Saldanha, 2015) or as neurohormones.

**TABLE 2:** Significant results of controlled endocrine studies investigating romantic love.

**Study**	**Factor**	**Findings**		
		**Women**	**Men**	**Women and men**
Marazziti et al., 1999	Serotonin transporter density			<* (normal controls)
Marazziti and Canale, 2004	Cortisol Testosterone FSH	> >	> < <	
Emanuele et al., 2006	NGF			> (relationship controls) > (single controls)
Langeslag et al., 2012	Serotonin	>	<	
Weisman et al., 2015	Cortisol			<
Marazziti et al., 2017	Dopamine transporter density and maximal velocity			<*
Sorokowski et al., 2019	Testosterone LH FSH	< > >		

<, lower level than control; >, higher level than controls; FSH, follicle-stimulating hormone; NGF, nerve growth factor; LH, luteinizing hormone; *, decreased transporter density is indicative of higher extracellular neurotransmitter levels.

Romantic love is associated with changes in the sex hormones testosterone, follicle-stimulating hormone, and luteinizing hormone (Marazziti and Canale, 2004; Durdiakova et al., 2017; Sorokowski et al., 2019), although the findings have been inconsistent. Testosterone appears to be lower in men experiencing romantic love than controls (Marazziti and Canale, 2004) and higher eros scores are associated with lower levels of testosterone in men (Durdiakova et al., 2017). Lower levels of testosterone in fathers are associated with greater involvement in parenting (see Storey et al., 2020, for review). The direction of testosterone change in women is unclear (see Marazziti and Canale, 2004; Sorokowski et al., 2019). Sex hormones are involved in the establishment and maintenance of sexual characteristics, sexual behavior, and reproductive function (Mooradian et al., 1987; Chappel and Howles, 1991; Holloway and Wylie, 2015). Some sex hormones can influence behavior through their organizing effects resulting from prenatal and postnatal exposure. In the case of romantic love, however, the effects of sex hormones on the features of romantic love are the result of activating effects associated with behaviorally contemporaneous activity. It is possible that sex hormones influence individual differences in the presentation of romantic love through their organizing effect (see Motta-Mena and Puts, 2017; Luoto et al., 2019; Arnold, 2020; McCarthy, 2020, for descriptions of organizing and activating effects of testosterone, estradiol, and progesterone). Changes in sex hormones could help to explain the increase in sexual desire and arousal associated with romantic love (Hatfield and Sprecher, 1986; Hatfield and Rapson, 2009; Diamond and Dickenson, 2012).

Romantic love is associated with decreased serotonin transporter density (Marazziti et al., 1999) and changes in plasma serotonin (Langeslag et al., 2012), although inconsistencies have been found in the direction of change according to sex. In one study, men experiencing romantic love displayed lower serotonin levels than controls and women displayed higher serotonin levels than controls (Langeslag et al., 2012). Decreased serotonin transporter density is indicative of elevated extracellular serotonin levels (Mercado and Kilic, 2010; Jørgensen et al., 2014). However, decreased levels of serotonin are thought to play a role in depression, mania, and anxiety disorders (Mohammad-Zadeh et al., 2008), including obsessive-compulsive disorder (for a discussion of the relationship between serotonin and OCD, see Baumgarten and Grozdanovic, 1998; Rantala et al., 2019). One study showed that a sample of mainly women (85% women) experiencing romantic love have similar levels of serotonin transporter density to a sample of both women and men (50% women) with obsessive-compulsive disorder (Marazziti et al., 1999), which could account for the intrusive thinking or preoccupation with the loved one associated with romantic love (Hatfield and Sprecher, 1986).

Lower dopamine transporter density and lower dopamine transporter maximal velocity in lymphocytes have been found in people experiencing romantic love (Marazziti et al., 2017). This is indicative of increased dopamine levels (Marazziti et al., 2017) and is consistent with neuroimaging studies (e.g., Takahashi et al., 2015; Acevedo et al., 2020) showing activation of dopamine-rich regions of the mesolimbic pathway. One study (Dundon and Rellini, 2012) found no difference in dopamine levels in urine in women experiencing romantic love compared with a control group. Dopamine is involved in reward behavior, sleep, mood, attention, learning, pain processing, movement, emotion, and cognition (Ayano, 2016). Up-regulation of the dopamine system could help to explain the motivational characteristics of romantic love such as longing for reciprocity, desire for complete union, service to the other, and maintaining physical closeness (Hatfield and Sprecher, 1986).

There are no studies that have specifically investigated oxytocin levels in romantic love (at least none that measure romantic love with a validated scale). However, studies (Schneiderman et al., 2012, Schneiderman et al., 2014; Ulmer-Yaniv et al., 2016) have demonstrated that people in the early stages of their romantic relationship have higher levels of plasma oxytocin than controls (singles and new parents). We infer this to mean that reciprocated romantic love is associated with elevated oxytocin levels. Oxytocin plays a role in social affiliation (IsHak et al., 2011) and pair-bonding (Young et al., 2011; Acevedo et al., 2020). Oxytocin receptors are prevalent throughout the brain including in the mesolimbic pathway (e.g., Bartels and Zeki, 2000). Elevated oxytocin could account for many of the behavioral features of romantic love such as actions toward determining the other’s feelings, studying the other person, service to the other, and maintaining physical closeness (Hatfield and Sprecher, 1986).

Romantic love has been associated with elevated cortisol levels (Marazziti and Canale, 2004), although this has not been replicated (Sorokowski et al., 2019), and one study measuring cortisol in saliva found the opposite (Weisman et al., 2015). Different results could be attributed to different length of time in a relationship between the samples (see Garcia, 1997; de Boer et al., 2012). Cortisol plays a role in the human stress response by directing glucose and other resources to various areas of the body involved in responding to environmental stressors while simultaneously deactivating other processes (such as digestion and immune regulation, Mercado and Hibel, 2017). Elevated cortisol levels may play a role in pair-bond initiation (Mercado and Hibel, 2017) and are indicative of a stressful environment.

Romantic love is associated with higher levels of nerve growth factor, and the intensity of romantic love correlates with levels of nerve growth factor (Emanuele et al., 2006). Nerve growth factor is a neurotrophic implicated in psycho-neuroendocrine plasticity and neurogenesis (Berry et al., 2012; Aloe et al., 2015; Shohayeb et al., 2018) and could contribute to some of the neural and endocrine changes associated with romantic love.

#### Insights From the Mechanisms of Psychopathology

Despite “madness” being mentioned in one review of the neurobiology of love (Zeki, 2007) and psychopathology being discussed in studies investigating the endocrinology of romantic love (e.g., Marazziti et al., 1999, 2017), the similarities between romantic love and psychopathology are under-investigated. An understanding of the mechanisms that regulate addiction, mood disorders, and anxiety disorders may help to shed light on the psychological characteristics and mechanisms underlying romantic love and identify areas for future research.

Conceptualizing romantic love as a “natural addiction” (e.g., Fisher et al., 2016) not only helps to explain romantic love’s psychological characteristics but provides insight into the mechanisms underlying it (e.g., Zou et al., 2016). For example, a neurocircuitry analysis of addiction, drawing on human and animal studies, reveals mechanisms of different “stages” of addiction that have implications for romantic love: binge/intoxication (encompassing drug reward and incentive salience), withdrawal/negative affect, and preoccupation/anticipation (Koob and Volkow, 2016). Each of these stages is associated with particular neurobiological activity and each stage could be represented in romantic love. This may mean that the findings of studies investigating the neurobiology of romantic love (which rely primarily on studies where visual stimuli of a loved one are presented) equates to the binge/intoxication stage of addiction. Findings from studies investigating romantic rejection (Fisher et al., 2010; Stoessel et al., 2011; Song et al., 2015) may equate to the withdrawal/negative affect stage of addiction. Findings from resting-state fMRI studies (Song et al., 2015; Wang et al., 2020) may equate to the preoccupation/anticipation stage of addiction. The result is that current neuroimaging studies may paint a more detailed picture of the neurobiology of romantic love than might initially be assumed.

Mood is an emotional predictor of the short-term prospects of pleasure and pain (Morris, 2003). The adaptive function of mood is, essentially, to integrate information about the environment and state of the individual to fine-tune decisions about behavioral effort (Nettle and Bateson, 2012). Elevated mood can serve to promote goal-oriented behavior and depressed mood can serve to extinguish such behavior (Wrosch and Miller, 2009; Bindl et al., 2012; Nesse, 2019). Anxious mood is a response to repeated threats (Nettle and Bateson, 2012). Because romantic love can be a tumultuous time characterized by emotional highs, lows, fear, and trepidation, and can involve sustained and repetitive efforts to pursue and retain a mate, it follows that mood circuitry would be closely intertwined with romantic love. Additionally, because romantic love concerns itself with reproduction, which is the highest goal in the realm of evolutionary fitness, it makes sense that mood may impact upon the way romantic love manifests. Understanding the mechanisms that regulate mood can provide insights into psychological characteristics of romantic love and the mechanisms that regulate it. No studies have directly investigated the mechanisms that contribute to changes in mood in people experiencing romantic love. However, insights can be taken from research into the mechanisms of mood and anxiety disorders.

While addiction, hypomania, depression, and anxiety symptoms in people experiencing romantic love may be the normal manifestation of particular mechanisms, symptoms associated with psychopathology may be the manifestations of malfunctioning mechanisms as a result of evolutionary mismatch (see Durisko et al., 2016; Li et al., 2018). As a result, the mechanisms that cause romantic love and those that cause psychopathology may not be precise models with which to investigate the other. Nonetheless, the mechanisms that cause psychopathology may provide a useful framework with which to base future research into romantic love. Conversely, it may also be that our understanding of the mechanisms that cause romantic love could be a useful framework with which to further investigate psychopathology.

##### Addiction

The drug reward and incentive salience features of the binge/intoxication stage of addiction involve changes in dopamine and opioid peptides in the basal ganglia (i.e., striatum, globus pallidus, subthalamic nucleus, and substantia nigra pars reticulata, Koob and Volkow, 2016). No research has investigated opioids in romantic love, despite them being involved in monogamy in primates (see French et al., 2018) and pair-bonding in rodents (Loth and Donaldson, 2021). The negative emotional states and dysphoric and stress-like responses in the withdrawal/negative affect stage are caused by decreases in the function of dopamine in the mesolimbic pathway and recruitment of brain stress neurotransmitters (i.e., corticotropin-releasing factor, dynorphin), in the extended amygdala (Koob and Volkow, 2016). No studies have investigated corticotropin-releasing factor in romantic love. The craving and deficits in executive function in the preoccupation/anticipation stage of addiction involve the dysregulation of projections from the prefrontal cortex and insula (e.g., glutamate), to the basal ganglia and extended amygdala (Koob and Volkow, 2016). No studies have investigated glutamate in romantic love. There are at least 18 neurochemically defined mini circuits associated with addiction (Koob and Volkow, 2016) that could be the target of research into romantic love. It is likely that romantic love has similar, although not identical, mechanisms to addiction (see Zou et al., 2016; Wang et al., 2020).

##### Mania/hypomania (bipolar disorder)

Similar to the brain regions implicated in romantic love, the ventral tegmental area has been associated with mania (Abler et al., 2008), the ventral striatum has been associated with bipolar disorder (Dutra et al., 2015), and the amygdala has been associated with the development of bipolar disorder (Garrett and Chang, 2008). These findings should be interpreted with caution, however, as replicating neuroimaging findings in bipolar disorder has proven difficult (see Maletic and Raison, 2014). Research implicates two interrelated prefrontal–limbic networks in elevated mood, which overlap with activity found in romantic love: the automatic/internal emotional regulatory network which includes the ventromedial prefrontal cortex, subgenual anterior cingulate cortex, nucleus accumbens, globus pallidus, and the thalamus, and the volitional/external regulatory network which includes the ventrolateral prefrontal cortex, mid- and dorsal-cingulate cortex, ventromedial striatum, globus pallidus, and thalamus (Maletic and Raison, 2014).

Norepinephrine (theorized to be involved in romantic love, e.g., Fisher, 1998, 2000), serotonin, dopamine, and acetylcholine play a role in bipolar disorder (Manji et al., 2003). One study (Dundon and Rellini, 2012) found no difference in norepinephrine levels in urine in women experiencing romantic love compared with a control group. No studies have investigated acetylcholine in romantic love but romantic love is associated with serotonin (Marazziti et al., 1999; Langeslag et al., 2012) and dopamine activity (Marazziti et al., 2017). Similar to the endocrine factors implicated in romantic love (Emanuele et al., 2006; Schneiderman et al., 2012, 2014; Ulmer-Yaniv et al., 2016), bipolar patients in a period of mania have also demonstrated higher oxytocin (Turan et al., 2013) and nerve growth factor (Liu et al., 2014) levels and lower levels of serotonin (Shiah and Yatham, 2000). Additionally, there is some evidence that women diagnosed with bipolar disorder present with higher levels of testosterone whereas men present with lower levels of testosterone compared with sex-matched controls (Wooderson et al., 2015). Similar findings have been found in romantic love (Marazziti and Canale, 2004). Dysfunction in the hypothalamic–pituitary–adrenal axis, where cortisol plays a major role, has also been implicated in bipolar disorder (Maletic and Raison, 2014). Cortisol probably plays a role in romantic love (Marazziti and Canale, 2004; Weisman et al., 2015).

##### Depression

Neuroimaging studies have implicated changes in functional connectivity in the neural circuits involved in affect regulation in people experiencing depression (Dean and Keshavan, 2017). Increased functional connectivity has been found in networks involving some of the same regions, such as the amygdala, the medial prefrontal cortex, and nucleus accumbens in both people experiencing romantic love and people who recently ended their relationship while in love (Song et al., 2015).

There are a number of endocrine similarities between romantic love and depression. One major pathophysiological theory of depression is that it is caused by an alteration in levels of one or more monoamines, including serotonin, norepinephrine, and dopamine (Dean and Keshavan, 2017). Altered dopamine transmission in depression may be characterized by a down-regulated dopamine system (see Belujon and Grace, 2017), which is inferred from numerous human and animal studies, including successful treatment in humans with a dopamine agonist. In romantic love, however, dopamine appears to be up-regulated, especially in areas of the mesolimbic pathway (e.g., Marazziti et al., 2017; Bartels and Zeki, 2000; Acevedo et al., 2020). This could account for some findings that romantic love is associated with a reduction in depression symptoms (Bajoghli et al., 2013, 2017). However, these need to be reconciled with contrasting findings that romantic love is associated with increased depression symptoms (Bajoghli et al., 2014; Kuula et al., 2020) and evidence suggesting that a relationship breakup in people experiencing romantic love is associated with depression symptoms (Stoessel et al., 2011; Price et al., 2016; Verhallen et al., 2019). The mechanisms that underlie depression might provide a framework for such efforts.

Dysregulation of the HPA axis and associated elevated levels of cortisol is theorized to be one contributor to depression (Dean and Keshavan, 2017). Changes in oxytocin and vasopressin systems (theorized to be involved in romantic love, e.g., Fisher, 1998, 2000; Carter, 2017; Walum and Young, 2018) are associated with depression (see Purba et al., 1996; Van Londen et al., 1998; Neumann and Landgraf, 2012; McQuaid et al., 2014). No studies have investigated vasopressin in people experiencing romantic love. There is also decreased neurogenesis and neuroplasticity in people experiencing depression (Dean and Keshavan, 2017), the opposite of which can be inferred to occur in romantic love because of its substantial neurobiological activity and elevated nerve growth factor (see Berry et al., 2012; Aloe et al., 2015; Shohayeb et al., 2018).

##### Anxiety

The insular cortex, cingulate cortex, and amygdala are implicated in anxiety and anxiety disorders (Martin et al., 2009). There is also evidence that cortisol, serotonin and norepinephrine are involved (Martin et al., 2009). The substantial overlap between the mechanisms regulating romantic love and those causing anxiety and anxiety disorders provides an opportunity to investigate specific mechanistic effects on the psychological characteristics of romantic love. Assessing state anxiety and these mechanisms concurrently in people experiencing romantic love may be a fruitful area of research.

There is also a need to clarify the role of the serotonin system in romantic love. Similar serotonin transporter density in platelets in people experiencing romantic love and OCD suggests a similar serotonin-related mechanism in both (Marazziti et al., 1999). However, lower serotonin transporter density in platelets is indicative of higher extracellular serotonin levels (Mercado and Kilic, 2010; Jørgensen et al., 2014). This is despite lower levels of serotonin being theorized to contribute to anxiety (Mohammad-Zadeh et al., 2008). One study found lower circulating serotonin levels in men experiencing romantic love than controls and higher levels of circulating levels of serotonin in women experiencing romantic love than controls (Langeslag et al., 2012). Insights from the mechanisms regulating anxiety disorders may help to provide a framework with which to further investigate the role of the serotonin system in romantic love and reconcile these findings.

#### Insights From Cognitive Biases

Positive illusions are cognitive biases about a relationship and loved one that are thought to have positive relationship effects (Song et al., 2019). The research into positive illusions does not use samples of people explicitly experiencing romantic love, and instead uses people in varied stages of a romantic relationship, including those in longer-term pair-bonds. One study (Swami et al., 2009), however, did find a correlation between the “love-is-blind bias” (one type of positive illusion) and eros scores. We also know that cognitive biases resembling positive illusions do exist in romantic love. Both the Passionate Love Scale (e.g., “For me, ____ is the perfect romantic partner,” Hatfield and Sprecher, 1986, p. 391) and the eros subscale of the Love Attitudes Scale (e.g., “My lover fits my ideal standards of physical beauty/handsomeness,” Hendrick and Hendrick, 1986, p. 395) include questions about a respondent’s loved one that resemble measures of positive illusions. Understanding the mechanism that regulates positive illusions will provide a model against which the mechanisms regulating the cognitive features of romantic love can be assessed.

A proposed mechanism of positive illusions includes the caudate nucleus, dorsal anterior cingulate cortex, ventral anterior cingulate cortex, orbitofrontal cortex, ventrolateral prefrontal cortical regions, and dorsal medial prefrontal cortex (Song et al., 2019). These regions overlap with the brain regions associated with romantic love. This suggests that the cognitive biases associated with romantic love may be related to, but are distinct from, positive illusions. Targeted neuroimaging studies could ascertain any involvement of the ventrolateral prefrontal cortex and the dorsal medial prefrontal cortex in romantic love. Such research could help to delineate a mechanism that specifically regulates one cognitive aspect of romantic love from those that regulate other psychological aspects of romantic love.

#### Insights From Mammalian Pair-Bonding Mechanisms

It is not possible to say with any certainty if other animals experience romantic love. Some certainly engage in pair-bonding and exhibit behaviors that are characteristic of romantic love such as obsessive following, affiliative gestures, and mate guarding (see Fisher et al., 2006). While some similarities between humans and other animals may be the result of parallel evolution, an understanding of the mechanisms involved in pair-bond formation in other animals can raise questions and guide research into romantic love in humans. Research into monogamous prairie voles, in particular, has identified neurobiological and endocrinological mechanisms that regulate pair-bonding processes. Drawing on this research, a hypothetical neural circuit model of pair-bond formation (pair-bonding) that includes the ventral tegmental area, nucleus accumbens, paraventricular nucleus, amygdala, hippocampus, anterior olfactory nucleus, and medial prefrontal cortex has been proposed (Walum and Young, 2018). Research implicates oxytocin, vasopressin, dopamine, and, potentially, serotonin and cortisol in pair-bonding (Walum and Young, 2018). Most of these neural regions and endocrine factors have been implicated in romantic love in humans. The implications of this research become apparent when the phylogeny of romantic love is presented.

### Ontogeny

When applied to romantic love, the second of Tinbergen’s (1963) four questions asks: “How does romantic love develop over the lifetime of an individual?” This can be answered with reference to the age of onset of romantic love, its presence throughout the lifespan, and its duration. Questions of ontogeny also encompass issues around the internal and external influences on romantic love (Tinbergen, 1963; Zeifman, 2001). We have also chosen to include some consideration of culture in this section because it influences the causes of romantic love. We find that romantic love first develops in childhood, is experienced at all ages in both sexes, usually lasts months or years, but can exist for many years or decades. It is influenced by a range of internal and external factors and is similar across cultures. The modern environment may influence romantic love in ways not present in our evolutionary history.

#### Romantic Love Over the Lifetime

Romantic love occurs from childhood through adulthood. It first manifests before puberty (Hatfield et al., 1988), with boys and girls as young as four reporting experiences that equate to romantic love. Adolescence is the time in which romantic love first manifests with all of its characteristic features (Hatfield and Sprecher, 1986), including the onset of sexual desire and activity and, potentially, pair-bonding. Romantic love may be more common among adolescents than young adults. In one study (Hill et al., 1997), American university psychology students reported a greater occurrence of mutual and unrequited love experiences when they were 16–20 years old compared to when they were 21–25 years old. However, romantic love exists at all ages of adulthood in both sexes (Wang and Nguyen, 1995).

There are few studies of psychological sex differences in romantic love. Those that exist (e.g., Hatfield and Sprecher, 1986; Hendrick and Hendrick, 1995; Cannas Aghedu et al., 2018) compare the overall intensity of romantic love and find no difference or slightly more intense romantic love in women than men. To our knowledge, no research has specifically investigated sex differences in duration or form of romantic love although it has been shown that some precursors to romantic love may play a greater role in one sex than the other (see Pines, 2001; Sprecher et al., 1994; Riela et al., 2010). As highlighted above, there are small sex differences in the neurobiology of romantic love (Bartels and Zeki, 2004; Fisher et al., 2006) and sex differences may exist in the activity of testosterone (Marazziti and Canale, 2004) and serotonin (Langeslag et al., 2012) in people experiencing romantic love, although findings have been inconsistent. These neurobiological and endocrinological differences may, presumably, have differential effects on the presentation of romantic love which have not yet been identified by research.

The psychological features of romantic love are said to normally last between 18 months to 3 years (Tennov, 1979), although studies have found that serotonin transporter density, cortisol levels, testosterone levels, follicle-stimulating hormone levels, and nerve growth factor levels do not differ from controls 12–24 months after initial measurement (Marazziti et al., 1999; Marazziti and Canale, 2004; Emanuele et al., 2006). Unrequited love has been shown to last an average duration of between 10 and 17 months, depending on the type of unrequited love (Bringle et al., 2013). In that study, unrequited love for someone that an individual pursued lasted the shortest period of time (10.12 months) and romantic love for someone who an individual knows but has not revealed their love to lasted the longest (18.44 months) in a sample of high school and university students from the United States. This contrasts with reciprocated romantic love that lasted even longer (an average of 21.33 months).

The early stages of romantic love characterized by stress may be distinct from a later period characterized by feelings of safety and calm (Garcia, 1997; de Boer et al., 2012). The first stage, which is characterized by approximately the first 6 months of a relationship, has been described as “being in love.” It is marked by all the characteristics of romantic love, including, especially, romantic passion and intimacy. The second phase, which has been said to last from approximately 6 months to 4 years, has been referred to as “passional love.” During this time passion is maintained but commitment and intimacy increase. Passional love gives way to companionate love, passion subsides, and commitment and intimacy reach their peaks. While a description of these phases is informative, it is important to recognize that only one study has investigated these phases and they used a sample of predominately university students (Garcia, 1997). Mechanisms research has not adopted these stages and “early stage” romantic love does not specifically refer to the first 6 months of a romantic relationship.

Romantic love exists on a continuum of intensity but can be classified categorically (Hatfield and Sprecher, 1986). The authors of the Passionate Love Scale (Hatfield and Sprecher, 2011) have developed arbitrary cutoffs for differing intensities of romantic love. However the thresholds that define them are not theoretically or empirically derived and are yet to be widely accepted in the literature.

Romantic love can commence abruptly or build up slowly, although the phenomenon of “love at first sight” may actually be strong attraction rather than romantic love, *per se* (Sternberg, 1986; Zsok et al., 2017). In one study of Chinese and American participants, 38% of participants fell in love fast and 35% fell in love slow, with the remaining unknown (Riela et al., 2010). Another study, of Iranians, found that 70% of participants fell in love slowly or very slowly (Riela et al., 2017). Romantic love can end abruptly but often wanes slowly.

Regardless of the normal duration of romantic love, there is a general inverse relationship between the length of time in a relationship and romantic love (Hatfield et al., 2008; Acevedo and Aron, 2009). Romantic love normally gives way to failure of a relationship to form, a relationship breakup, or transition to companionate love. However, in some individuals, romantic love can last many years, or even, decades (O’Leary et al., 2011; Acevedo et al., 2012; Sheets, 2013). In romantic relationships that last, romantic love serves to bond partners together by creating shared understandings, emotions, and habits (Hatfield and Walster, 1985) characteristic of companionate love and long-term pair-bonds. The transition from romantic love to companionate love is gradual and both types of love share many characteristics. In circumstances where romantic love is maintained beyond the initial few years, obsessive thinking about a partner is no longer a feature (e.g., Acevedo and Aron, 2009; O’Leary et al., 2011).

#### Internal and External Influences

A number of internal and external influences affect when, with whom, and how we fall in love. The scenario of attachment, separation, and loss in young children (Bowlby, 1969, 1973, 1980) is similar to a “desire for union” and may be the groundwork for romantic attachments in later life (Hatfield et al., 1988). To this extent, romantic love, like newborn/infant attachment, is “prewired” into humans as part of their evolutionary heritage (Hatfield et al., 1989). Researchers “focus their investigations on the effects of mother-infant bonding in order to explain variations in the form, duration, and/or frequency of adult passionate relationships” (Fisher, 1998, p. 31). For example, a person’s adult attachment style is determined in part by childhood relationships with parents (Hazan and Shaver, 1987) and this may have implications for the experience of romantic love (e.g., Hendrick and Hendrick, 1989; Aron et al., 1998). Romantic love is positively associated with a secure attachment style and negatively associated with an avoidant attachment style.

Precursors to romantic love include reciprocal liking, appearance, personality, similarity, social influence, filling needs, arousal, readiness, specific cues, isolation, mysteriousness, and propinquity (see Aron et al., 1989; Sprecher et al., 1994; Riela et al., 2010; Riela et al., 2017; see also Hazan and Diamond, 2000; Fisher, 2011). Research also suggests that conscious variables (personality and appearance), situational variables (proximity and arousal), lover variables (lover finds us attractive, lover fills important needs, similarity, and lover is best friend), and unconscious variables (similarity to relationship with parents, similarity of lover to father, similarity of lover to mother, and love at first sight) contribute to with whom we fall in love (Pines, 2005). The majority of precursors are an interplay between internal and external influences.

Some of the most important precursors to romantic love include personality, reciprocal liking, physical appearance, propinquity, specific cues, readiness, and similarity (Aron et al., 1989; Sprecher et al., 1994; Riela et al., 2010, 2017). Personality is the “attractiveness of the other’s personality (e.g., intelligent, humorous)” (Riela et al., 2010, p. 474). This represents an interplay between internal influences (the preferences of the individual or what they find attractive) and external influences (the personality characteristics of the potential loved one). Reciprocal liking has been defined above and is a mixture of internal and external influences. Physical appearance, too, is an interplay between what an individual finds attractive, either through genetic predisposition or learned experience, and the physical attributes of the potential loved one. Propinquity has been defined and discussed above and is a combination of internal and external influences. Similarity is “having things in common, including attitudes, experiences, interests, and personal factors such as appearance, personality, and family background (Riela et al., 2010, p. 474). This is contingent upon both the individual’s characteristics (internal influence) and the potential loved one’s characteristics (external influence).

There are, however, some precursors that are explicitly internal or external influences. Readiness is “being emotionally or physically prepared for seeking a romantic relationship, such as having just broken up with someone and seeking comfort in a new partner” (Riela et al., 2010, p. 475). This can be a largely internal influence that can cause romantic love. Specific cues are “particular characteristics of the other (e.g., smile, shape of the eyes), that are relevant to the perceiver in producing strong attractions. This is not the same as attractiveness in general but refers to highly idiosyncratic features of potential love objects that are specifically important to the individual” (Riela et al., 2010, p. 475). These are largely external influences that cause romantic love, although they do trigger a biological or psychological response which is internally determined.

#### Cross-Cultural Perspectives

There have been a number of books (e.g., Jankowiak, 1995, 2008) and studies that shed light on the cross-cultural nature of romantic love. The sum of research indicates that romantic love is probably universal (although the research is yet to prove this unequivocally) with relatively few psychological differences found between cultures (although cultures respond to love in different ways). An ethnographic analysis of 166 cultures from the Standard Cross-Cultural Sample (Jankowiak and Fischer, 1992; Jankowiak and Paladino, 2008) found no evidence of romantic love in only 15 cultures, and this was largely due to lack of data. Validated measures of romantic love (i.e., Passionate Love Scale, Love Attitudes Scale, Triangular Love Scale) have been used in at least 50 countries (Feybesse and Hatfield, 2019). The Triangular Theory of Love is robust cross-culturally (Sorokowski et al., 2020). Cross-cultural accounts of the features and the intensity of romantic love are remarkably similar (see Feybesse and Hatfield, 2019 for a review of cross-cultural perspectives on romantic love). Multiple neuroimaging studies have ascertained that the same neural mechanisms associated with romantic love in American samples are associated with romantic love in Chinese samples (Xu et al., 2011, 2012b).

Romantic love may be thought of more positively among Western countries than other countries and Westerners report falling in love more often (see Feybesse and Hatfield, 2019). Cultural differences have also been identified in the role of precursors in causing romantic love. A comparison between Japanese, Russian, and American populations found that culture played a role in the self-reported importance of personality, physical appearance, propinquity, similarity, readiness, isolation, mystery, and social standing (Sprecher et al., 1994). Some differences have also been found between Chinese and Americans (Riela et al., 2010) and between Iranians and Americans (Riela et al., 2017) using similar and different methods. In some cultures, romantic love is suppressed and arranged marriages predominate (discussed below).

#### Evolutionary Mismatch

The evolutionary mismatch hypothesis argues that humans are now living in environments vastly different from those in which they evolved and, as a result, biological mechanisms may not interact with the environment in the manner that they originally evolved to Li et al. (2018). Adaptations may malfunction. This has implications for the functioning of mechanisms and psychology. Evolutionary mismatch may influence the occurrence, duration, form, and experience of romantic love. As already suggested, evolutionary mismatch may influence the degree to which certain social mechanisms play a role in causing romantic love. This may have flow-on impacts on the frequency with which an individual falls in love or with whom they fall in love. The increased exposure to potential mates may also lead to greater instances of relationship dissolution and new instances of romantic love than would have been the case in our evolutionary history. Evolutionary mismatch may also influence the duration of romantic love. Under evolutionary conditions, romantic love would usually occur in the context of reproduction, pregnancy, and childbirth (see Goetz et al., 2019). This may mean that the duration of romantic love may have been shorter in females than is the case in modern developed societies because they are overcome by mother-infant bonding, possibly at the expense of romantic love.

The form and experience of romantic love may also be impacted by evolutionary mismatch. Technology means that lovers are able to maintain regular contact (e.g., by telephone) or be exposed to images of the loved one (e.g., by photographs) in the absence of physical contact. This consistent exposure may be associated with more frequent activation of neural structures associated with romantic love (i.e., reward and motivation structures) and change the intensity or subjective experience of romantic love compared to evolutionary ancestors who may have been completely separated for periods of time.

## Ultimate Perspectives

### Functions

When applied to romantic love, the third of Tinbergen’s (1963) four questions asks: “What are the fitness-relevant functions of romantic love?” Functional explanations address the fitness ramifications (survival and reproduction) of the behavior or trait of interest (Tinbergen, 1963; Zeifman, 2001; Bateson and Laland, 2013). We are, thus, concerned with both the fitness-relevant benefits and costs of romantic love. We have outlined the benefits and costs of romantic love associated with five functions based on a small literature on the subject (i.e., Fletcher et al., 2015; Buss, 2019), reproduction-related literature, and our consideration of the subject. Some of the benefits we describe can be considered functions in their own right (e.g., Buss, 2019). Table 3 presents a summary of benefits and costs of romantic love according to five distinct yet interrelated functions: mate choice, courtship, sex, pair-bonding, and health. Our approach is to describe each function, present the benefits associated with each function, and present the costs associated with each function. Where relevant, we have included information about related concepts or theories. We contend that while there is a small amount of evidence for the health promoting benefits of romantic love, the evidence is insufficient to say with certainty that health promotion is a function of romantic love. We conclude this section by summarizing some potential selective pressures and describing romantic love as a complex suite of adaptations and by-products.

**TABLE 3:** Reproduction- and survival-related benefits and costs associated with each function of romantic love.

	**Benefits**	**Costs**
Reproduction-related:		
Mate choice	Conserve mating energy, choose between potential mates, focus attention on preferred mates (♀/♂)	Imperfect mate choice, excluding other potential mates, detract from other goals, unwanted love experience (♀/♂)
Courtship	Pursue potential mates, secure a mate prepared to commit, display commitment, signal fidelity, learn about and assess potential mates, display reproductively relevant resources (♀/♂); Signal paternal investment (♂)	Expenditure of time and resources, embarrassment, obsessive pursuit, stress, intrasexual competition, costly courting (♀/♂)
Sex	Reputation and status gain, sex is pleasurable, sex promotes bonding (♀/♂); Providing sexual access, increased fecundity (possibly) (♀); Gaining sexual access (♂)	Unwanted pregnancy, parenting responsibilities, damage to reputation and status (♀/♂), Pregnancy followed by a period of lactation, risk of single parenthood (♀)
Pair-bonding	Establish pair-bonds, provision of psychological and emotional resources, caregiving, promote fidelity, promote jealousy, promote relationship exclusivity through mate guarding, promote mate retention tactics, sharing resources, reputation and status gain, increased offspring survival (possibly), promote fitness interdependence, promote self-expansion (♀/♂); Paternal investment (♀); Promote actions that lead to successful reproductive outcomes, co-parenting (♂)	Missed long-term mating opportunities, restricted short-term mating opportunities, damage to reputation and status, sharing of time and resources, reduced support network, jealousy, harmful relationships, homicide, stalking, grief following breakup, other breakup costs, (♀/♂); Sexual obligation to partner (♀); Parental investment (♂)
Survival-related:		
Health	Active/elated mood, reduced depression symptoms, decreased risk of STI, improved sleep quality (♀/♂); Stronger immune system (♂)	STI, negative mood, major depression, suicide (♀/♂); Sleep alterations, birth-related complications/death, infertility from STI (♀)

*STI, sexually transmitted infection.*

#### Mate Choice

Romantic love serves a mate choice function (Fisher et al., 2006). Both men and women engage in mate choice (Stewart-Williams and Thomas, 2013). Assessing potential mates has important fitness consequences for individuals, as the benefits of finding a suitable mate are often higher than mating haphazardly or with a randomly selected mate (Geary et al., 2004; Andersson and Simmons, 2006; Jones and Ratterman, 2009; Shizuka and Hudson, 2020). On the other hand, mate choice is a costly and error-prone activity and, thus, it may be adaptive to focus one’s attention on a particular mate that has been identified as a preferred partner (Bowers et al., 2012). Romantic love serves this function.

Mate choice evolved in mammals to enable individuals to conserve their mating energy, choose between potential mates, and focus their attention on particular potential mating partners (Fisher, 2000; Fisher et al., 2006). The focus of one’s attention on a single potential mate is not without costs (e.g., Klug, 2018; Bear and Rand, 2019). Imperfect mate choice (e.g., Johnstone and Earn, 1999) could result from imperfect information (e.g., Luttbeg, 2002) or acceptance or rejection errors. Imperfect information might include the concealment of information that has detrimental effects on fitness. Time to assess an individual is important in mate choice and imperfect mate choice could potentially be a greater problem in circumstances where romantic love is quick to arise. Mate choice, by definition, excludes other potential mates and romantic love, in fact, suppresses the search for other mates (Fletcher et al., 2015). This cost can be exacerbated in certain environments such as those within which finding additional mates is relatively easy (Kushnick, 2016). Romantic love can detract from other fitness-promoting goals such as career-advancing activities, physical health promoting activities, or forming and maintaining other social relationships.

#### Courtship

Romantic love serves a courtship function (Fisher et al., 2006, 2016). Courtship involves a series of signals and behaviors that serve as a means of assessing potential partner quality and willingness to invest in a relationship (Trivers, 1972; Wachtmeister and Enquist, 2000). One function of the attraction system is to pursue potential mates (Fisher, 2000). People in love often engage in courtship of their loved one with the aim of persuading them that they are a good long-term mate.

The primary benefit of courtship in romantic love is that it can secure a mate that is prepared to commit to a relationship. To do this, both sexes can pursue potential mates, display commitment, and signal fidelity (Buss, 2019). These acts are why love has been described as a commitment device (Frank, 1988; Fletcher et al., 2015; Buss, 2019). Courtship allows individuals to learn about and assess the suitability of potential mates while displaying reproductively relevant resources (Buss, 2019). Men emphasize characteristics such as resources, while women emphasize characteristics such as beauty, in an attempt to increase attractiveness (Buss, 1988; Luoto, 2019a). Men, at least historically, also provide signals of parental investment (Buss, 2019). Literature on human courtship from an evolutionary perspective supports the notion of greater choosiness among females, predicted by parental investment theory (Trivers, 1972), for short-term mating and less serious commitments. This effect, however, substantially diminishes for long-term mating endeavors and marriage commitment (Kenrick et al., 1990). The literature also suggests that women are looking for specific cues, indicative of evolved preferences, during the courtship process (Oesch and Miklousic, 2012).

There are costs associated with romantic love’s courtship function. These include the expenditure of a significant amount of time and resources and, if courtship efforts are not reciprocated, embarrassment (Silver et al., 1987). Sometimes, individuals in love might engage in intrusive “obsessive pursuit” of someone who is not interested (Spitzberg and Cupach, 2003). Courtship can be a particularly stressful time for an individual. There are also potential costs because individuals who are courting might find themselves in direct intrasexual competition with another individual who has an interest in their potential mate. Intrasexual competition can be costly because an individual must divert additional resources to this endeavor. An individual bears even greater costs if they lose this competition. Both sexes can be subject to costly signaling as part of courtship (Griskevicius et al., 2007), although men are at risk of higher fitness costs associated with temporally extended courtships, despite this being interpreted as a sign of a good mate by women (Seymour and Sozou, 2009).

#### Sex

Romantic love promotes sex and may increase the chances of pregnancy. Sex is an important part of romantic relationships and initiation into sex with a partner, and a greater frequency of sex, is associated with the earlier stages of a romantic relationship (Call et al., 1995). Sex and pregnancy are not, however, features of romantic love in pre-pubescent children and pregnancy is not a feature of romantic love in post-menopausal women. The nature of reproduction is different in societies where contraception and family planning practices are widespread (see Goetz et al., 2019, for review of evolutionary mismatch in human mating). In such circumstances, immediate pregnancy may not be a feature of romantic love, whereas sex often is. In such circumstances, romantic love may indirectly promote pregnancy by creating pair-bonds whose members later reproduce.

Romantic love provides sexual access (Buss, 2019). Love is one of the most common reasons people give for having sex (Ozer et al., 2003; Meston and Buss, 2007; Dawson et al., 2008; Meston and Buss, 2009). Given the relative willingness of men to engage in short-term mating compared to women, it follows that sex because of love plays a greater role in providing sexual access by women to men than the other way around (Meston and Buss, 2007). Sex can facilitate a gain in reputation (Meston and Buss, 2007) and both sexes increase their status by having children (Buss et al., 2020). Sex is intrinsically pleasurable and reinforcing, and promotes bonding (Meltzer et al., 2017). In times before the advent of contraception, repeated sex with a partner would usually result in pregnancy and childbirth (Goetz et al., 2019; Kushnick, 2019). This is still the case in many parts of the world.

For example, there is evidence that features characteristic of romantic love may be associated with a greater number of children among the Hadza, a hunter gatherer tribe in northern Tanzania (Sorokowski et al., 2017). Higher passion, which is definitive of romantic love (e.g., Sternberg, 1986), is associated with a greater number of children in women. The findings are important because the lifestyle of the Hadza more closely resembles the environment in which humans evolved than do industrialized or agrarian societies. As a result, inferences can be made about the adaptive function of passion in human evolutionary history. However, intimacy, another component of romantic love (Sternberg, 1997), was found to be negatively correlated with number of children in women. Instead, commitment, a feature of companionate love, was associated with greater number of children in both women and men (Sorokowski et al., 2017). Romantic love is normally relatively short-lived, and therefore the methods used in this study may not have been ideally suited to investigate the fitness consequences of romantic love. Nonetheless, this finding provides some support for the notion that romantic love promotes sexual access by women and facilitates reproduction.

One study (Sorokowski et al., 2019) suggests that romantic love may increase the likelihood of a woman falling pregnant. Higher levels of the gonadotropins, follicle-stimulating hormone, and luteinizing hormone, and a non-significant but positive increase in estradiol to testosterone ratio in women experiencing romantic love could cause increased ovarian activity and increased estradiol synthesis, which might result in higher fecundity (Sorokowski et al., 2019).

The costs associated with romantic love’s sex function are far greater for women than for men (Trivers, 1972). Both sexes could be subject to unwanted pregnancy and associated parenting responsibilities (although this impacts women to a greater extent). There is also, however, a risk of damage to an individual’s reputation. Women are often subject to criticism from other women for engaging in sexual activity (Koehn and Jonason, 2018), especially if a long-term relationship does not result. Men and women risk damage to their reputation for having sex with a low mate value partner, although men are generally treated far more favorably than women for engaging in sexual activity (see Zaikman and Marks, 2017). For women, a period of pregnancy followed by a lengthy period of lactation may ensue, and this is costly in terms of the ability to obtain sufficient resources and protecting oneself from harm. There is also the possibility that the relationship will dissolve following pregnancy and the woman may be left to raise a child without the father’s support (Koehn and Jonason, 2018).

#### Pair-Bonding

Romantic love serves a pair-bonding function (Fletcher et al., 2015). Pair bonding is both a process and a sate characterized by the formation of “enduring, selective attachments between sexual partners” (Young et al., 2011, p. 1). It differs from established pair-bonds and the neural characteristics of people experiencing romantic love differ somewhat from what is associated with longer-term pair-bonds (see Acevedo et al., 2012, for distinction). Evolutionarily, when sex more often led to pregnancy, this pair-bonding would occur in the context of pregnancy and childbirth (although it is unclear if romantic love can exist at the same time as mother-infant bonding). This is still the case in many parts of the world. This is one possible reason for the duration of reciprocated romantic love to be between 18 months and 3 years (Tennov, 1979) when not interrupted by childbirth. The intensity of specific neural activity in people experiencing romantic love is associated with relationship maintenance (Xu et al., 2012a).

Romantic love can establish long-term pair-bonds. In both sexes, romantic love promotes the provision of psychological and emotional resources (Buss, 2019) as well as other types of caregiving (Fletcher et al., 2015). It promotes relationship exclusivity through fidelity, jealousy, and mate-guarding (Buss, 2019). Both sexes engage in additional mate retention tactics such as vigilance, mate concealment, monopolization of time, resource display, love and care, or sexual inducements (Buss et al., 2008). Romantic love also promotes the sharing of other resources such as food or money. This benefit for women would have been, and often continues to be, greatest during times of lactation (see Marlowe, 2003; Quinlan, 2008). Both sexes can also benefit reputationally, as being in a relationship with a high mate value individual confers status, and individuals who are married or in a relationship are viewed more favorably than single people (DePaulo and Morris, 2006). Men experiencing romantic love engage in actions that lead to successful reproductive outcomes (Buss, 2019), such as protecting partners from physical harm. Men also engage in parenting (Geary et al., 2004; Bribiescas et al., 2012), which could potentially result in increased offspring survival (Fletcher et al., 2015).

When people are experiencing romantic love they are usually, but not always, interested in pursuing a “long-term mating strategy.” A long-term mating strategy is one that involves commitment, pair-bonding, and the parental investment (if children result) of both partners (Buss, 2006). This contrasts with short-term mating strategies that do not often require public commitment, pair-bonding, and parental investment of the father (Buss and Schmitt, 1993). Pair-bonding is characteristic of a long-term mating strategy.

The concept of romantic love serving as a commitment device is relevant to pair-bonding, as are the concepts of fitness interdependence (Buss, 2019) and self-expansion. Fitness interdependence is the degree to which two people influence each other’s success in replicating their genes (Aktipis et al., 2018). Romantic love binds two individuals together so that the potential reproductive success of one person is contingent upon the success of the other. The self-expansion model suggests that “people seek to expand their potential efficacy to increase their ability to accomplish goals” and that “one way people seek to expand the self is through close relationships, because in a close relationship the other’s resources, perspectives, and identities are experienced, to some extent, as one’s own” (Aron and Tomlinson, 2019, p. 2). Fitness interdependence and self-expansion can be increased in people experiencing romantic love.

There are substantial costs associated with pair-bonding (Kushnick, 2016; Klug, 2018). Both sexes are potentially missing out on long-term mating opportunities with other suitable mates and are more restricted in terms of short-term mating opportunities (Geary et al., 2004). There is a potential for damage to an individual’s reputation if they are in a relationship with a low mate value individual (Buss, 2016). Both sexes share resources. Pair-bonding is associated with a reduction in the size of an individual’s support network (Burton-Chellew and Dunbar, 2015). Jealousy can have negative effects upon a relationship (Buss, 2000, 2019; Hatfield et al., 2016) and there is a potential for emotional or physical harm arising from a relationship. People sometimes engage in homicide of their current or former partners in response to infidelity, or as a result of jealousy or a breakup (Buss, 2000, 2019; Shackelford et al., 2003). Some women engage in this behavior, but it is predominately a male behavior, when it occurs (Buss, 2019). Stalking can occur following a breakup (Spitzberg and Cupach, 2003; Buss, 2019) or, more generally, as a result of romantic love (Marazziti et al., 2015). There is the potential for grief or depression symptoms following the breakup of a relationship (Verhallen et al., 2019). Changing living arrangements, dividing up resources, and legal costs could all be necessary following the dissolution of a pair-bond (Bear and Rand, 2019). Sex-specific costs include sexual obligations to a partner from women and parental investment by men (Geary et al., 2004; Luoto, 2019a).

#### Health

While there is evidence that successful pair-bonding is associated with better health and survival (Fletcher et al., 2015), there is little evidence showing that romantic love is associated with good health. Falling in love is associated with alteration in immune cell gene regulation in young women (Murray et al., 2019). Specifically, falling in love is associated with genetic changes that could potentially result in an up-regulation of immune responses to viruses.

Experiencing romantic love for a recently gained partner is associated with the “active/elated” symptoms of hypomania (Brand et al., 2007, 2015). These symptoms are considered as favorable, “bright side” symptoms and contrast with unfavorable “dark side” symptoms such as disinhibition/stimulation-seeking and irritable/erratic dimensions (Brand et al., 2015). Despite their association with hypomania, the favorable nature of these symptoms in romantic love may be a sign of good physical and mental health because higher hypomanic scores have been associated with higher “mental toughness,” increased physical activity, lower symptoms of depression, and lower sleep complaints (Jahangard et al., 2017). Additionally, falling in love with a partner is sometimes associated with a reduction in depressive symptoms (Bajoghli et al., 2013, 2017). A reduction in the number of sexual partners could result in a decreased risk of sexually transmitted infections. There is evidence that romantic love might sometimes be associated with improved sleep quality (Brand et al., 2007; Bajoghli et al., 2014).

There are some health-related costs associated with romantic love for both sexes. Despite a reduced risk of sexually transmitted infections being a benefit of romantic love, engaging in sexual activity at all may represent an increased risk of sexually transmitted infection, resulting in a cost to some (Buss, 2016; Koehn and Jonason, 2018). Infertility from sexually transmitted infections is possible among women (Koehn and Jonason, 2018). Disinhibited/stimulation-seeking and irritable/erratic, depressed, and anxious mood are sometimes features of romantic love (Wang and Nguyen, 1995; Bajoghli et al., 2013, 2014, 2017; Brand et al., 2015; Kuula et al., 2020). In the face of repeated unrewarding efforts or adverse events in the courtship process, depressed or anxious mood could result (Nettle and Bateson, 2012). Romantic rejection can result in a major depressive episode or even suicide (see Rantala et al., 2018). Despite evidence of improved sleep quality in people experiencing romantic love in some studies (Brand et al., 2007; Bajoghli et al., 2014), one study (Kuula et al., 2020) found poorer sleep quality, later sleep timing, and shorter sleep duration (one feature commonly found in studies relied upon to suggest a sleep quality benefit of romantic love) in adolescent girls experiencing romantic love. This suggests that altered sleep may in fact be a detrimental cost in some people experiencing romantic love. Women have the added risk of birth-related complications and death, which has been common in humans until recently in developed countries (Goldenberg and McClure, 2011).

#### Selective Pressures

The literature contains three interesting theories of possible selective pressures for romantic love. They are framed in the context of promoting the evolution of pair-bonds, but as will be detailed below, the evolution of pair bonds and romantic love are likely to be inexorably linked. All three theories relate to the provision of resources by males to females. The first theory is that pair-bonds and romantic love may have emerged prior to 4 million years ago when bipedalism emerged and hominins moved into the woodlands and savannahs of our ancestral homelands (see Fisher et al., 2016). The need for mothers to carry infants in their arms may have driven them to select partners that were wired for pair-bonds which was associated with provisioning, defense, and other forms of support.

The second theory is that biparental care was a driving force in the emergence of long-term mating strategies (Conroy-Beam et al., 2015). A game theoretical approach contends that females selecting males that were wired for pair-bonds directly increased the chances of offspring survival through the provisioning of tangible and intangible resources to the female and offspring. If biparental care was a driving force in the formation of pair-bonds in humans, it would be a uniquely human pressure, as biparental care has been generally identified as a consequence, rather than a cause, of pair-bonds in mammals (Opie et al., 2013; Lukas and Clutton-Brock, 2013). This theory also has to contend with the fact that father presence is often not associated with better offspring survival in societies with little access to health care or contraception (see Fletcher et al., 2015).

The third theory is that a need for increased fecundity drove the selection of pair-bonds (Conroy-Beam et al., 2015). Periods of malnutrition cause decreased fecundity. Once again, a game theoretical approach suggests that the selection of males that were wired for pair-bonds, which is associated with provisioning of females, increased the caloric intake of females over prolonged periods of time and, in turn, increased fecundity. This hypothesis is appealing because this selective pressure could have been present at any stage among the four hypotheses we propose for the emergence of pair-bonds in a section below.

#### Romantic Love Is a Complex Suite of Adaptations and By-Products

In evolutionary psychology, an adaptation is “…an inherited and reliably developing characteristic that came into existence as a feature of a species through natural selection because it helped to directly or indirectly facilitate reproduction during the period of its evolution” (Buss et al., 1998, p. 535; see also Williams, 2019). This approach is based, rightly, on the difficulty of testing hypotheses about the adaptive benefits of traits in ancestral environments. There is an equally valid approach, however, adopted by behavioral ecologists, that views current utility of adaptations as evidence that can be extrapolated to the past (Fox and Westneat, 2010). One definition that has arisen from this approach is that “[a]n adaptation is a phenotypic variant that results in the highest fitness among a specified set of variants in a given environment” (Reeve and Sherman, 1993, p. 9).

Taken together, these two approaches to adaptation support the view that romantic love is a “complex suite of adaptations” (Buss, 2019, p. 42). The numerous mechanisms recruited in romantic love, the large number of psychological characteristics, and the multiple functions it serves suggest that romantic love may be an amalgamation of numerous adaptations that respond to a variety of adaptive challenges. However, while romantic love may comprise several inter-related adaptations, this does not preclude the possibility that some components are by-products. A by-product is a trait that evolved “not because it was selectively advantageous, but because it was inextricably linked…to another trait that was reproductively advantageous” (Andrews et al., 2002, p. 48).

Health-promoting benefits of romantic love, such as elevated mood, increased sleep quality, and up-regulated immune responses, for example, may be by-products of mood circuitry (see Nettle and Bateson, 2012; D’Acquisto, 2017; Jahangard et al., 2017) or other mechanisms, even though they offer some survival or reproductive advantage. Elevated mood, better sleep quality, and an associated up-regulated immune system probably evolved prior to the emergence of romantic love (see Flajnik and Kasahara, 2010; Loonen and Ivanova, 2015). As a result, it might be prudent to contend that romantic love is a complex suite of adaptations and by-products.

Further, while the evidence points to romantic love as a suite of adaptations and by-products, it is not adaptive in every context. Romantic love continues to have its reproduction-promoting functions in the modern world in some circumstances, either by immediately promoting reproduction, or indirectly promoting reproduction via the formation of romantic relationships, the members of which later reproduce. To that extent, romantic love is sometimes adaptive (see Laland and Brown, 2011, for distinction between “adaptation” and “adaptive” and lists of benefits, above, for examples of how romantic love can be adaptive). There are circumstances when romantic love may be maladaptive, however, as is evidenced by the substantial fitness-relevant costs of romantic love detailed above. Cogent examples of this are when a loved one is already in a committed relationship or otherwise not interested, when an individual engages in obsessive pursuit that can have social or even legal ramifications, or when violence ensues.

### Phylogeny

When applied to romantic love, the fourth of Tinbergen’s (1963) four questions asks, “What is the evolutionary history of romantic love?” Phylogenetic explanations focus on the origin and maintenance of a trait in historical evolutionary terms (Tinbergen, 1963; Bateson and Laland, 2013). They put a biological trait in a comparative perspective by focusing on the presence or absence of the trait in closely, and sometimes more distantly, related species. In this section, we describe the theory of independent emotion systems and articulate a theory of co-opting mother-infant bonding mechanisms. We examine the primitive structures related to romantic love that arose in our mammalian evolutionary past and were restructured in pair-bonded species. We also examine the particular history of pair-bonds, and thus romantic love, in hominin evolution, with a comparison to other species of primates, especially apes. Finally, we examine the effect of gene-cultural evolutionary issues with regard to romantic love.

#### Independent Emotion Systems

Fisher’s (1998, 2000, see also Fisher et al., 2002) evolutionary theory of independent emotions systems delineates sex drive (lust), attraction (romantic love), and attachment (pair-bonds). Sex drive is primarily associated with estrogens and androgens and serves to motivate individuals to engage in sexual activity, generally. Attraction is primarily associated with the catecholamines (i.e., dopamine and norepinephrine), phenylethylamine, and serotonin and serves to focus efforts on preferred mating partners. Attachment is primarily associated with oxytocin and vasopressin and serves to enable individuals to engage in positive social behaviors and connections of a sufficient length of time to satisfy species-specific parenting approaches (Fisher, 1998). Sex drive relates most to the sex function of romantic love, attraction to the mate choice and courtship functions, and attachment to the pair-bonding function. Romantic love shares similarities with the ‘courtship attraction system’ found in many mammals (Fisher et al., 2006).

#### Co-opting Mother Infant Bonding Mechanisms

While the theory of independent emotion systems (Fisher, 1998, 2000; Fisher et al., 2002) has been the predominate theoretical account of the evolution of romantic love for more 20 years, comparative studies, imaging studies, and assessments of psychological characteristics have raised the possibility of a complimentary evolutionary theory, that of co-opting mother-infant bonding mechanisms. Literature on romantic love, maternal love (of which mother-infant bonding is a part), mother–infant bonding, and pair-bonding (Bartels and Zeki, 2004; Ortigue et al., 2010; Numan and Young, 2016; Walum and Young, 2018) suggests romantic love may have evolved by co-opting mother-infant bonding mechanisms. Co-option is an evolutionary process whereby a trait (e.g., mechanism, morphology, behavior) is repurposed – that is, it serves a different function to that which it originally served (see McLennan, 2008).

Animal research, focusing on mammals, and involving, monogamous prairie voles, finds substantial similarities between mother-infant bonding mechanisms and pair-bonding mechanisms (Numan and Young, 2016). “[A]mygdala and nucleus accumbens–ventral pallidum (NA–VP) circuits are involved in both types of bond formation, and dopamine and oxytocin actions within NA appear to promote the synaptic plasticity that allows either infant or mating partner stimuli to persistently activate NA–VP attraction circuits, leading to an enduring social attraction and bonding” (Numan and Young, 2016, p. 98). Some of these circuits do not appear to be involved in human romantic love, but there are other similarities that support a theory of co-opting mother-infant bonding in humans.

Several brain regions implicated in romantic love overlap precisely with that involved in maternal love. This includes activity in numerous regions that are associated with a high density of oxytocin and vasopressin receptors (Bartels and Zeki, 2000, 2004) although it should be noted that in the study that asserts this, participants included mothers experiencing maternal love beyond the mother-infant bonding stage. A meta-analysis of love also found romantic and maternal love shared activity in dopamine-rich areas (Ortigue et al., 2010). Almost nothing is known about the mechanisms regulating the infant side of mother-infant bonding. However, some inferences have been made from animal models which suggest that the mechanisms may be similar to those regulating the maternal side, but without involvement of the amygdala (see Sullivan et al., 2011, for review).

There are substantial psychological similarities between romantic love and early parental love, of which mother–infant bonding is a part. Extreme similarities exist between romantic love and early parental love in the domains of altered mental state, longing for reciprocity, idealization of the other, and dichotomous resolution of the establishment of intimate mutually satisfying reciprocal patterns of interaction usually marked by a culturally defined ritual (Leckman and Mayes, 1999). Similar trajectories of preoccupation in romantic love and parental love also exist. In romantic love, preoccupation increases through the courtship phase and peaks at the point of reciprocity where preoccupation begins to slowly diminish. In parental love, preoccupation increases throughout pregnancy and peaks at the point of birth where preoccupation begins to diminish.

#### Mammalian Antecedents

Romantic love in humans is caused by physiological mechanisms whose evolutionary roots were planted in our early mammalian ancestors. These evolutionary roots provided the raw materials that were fleshed out, in evolutionary time, to form the basis of a wide range of social behaviors in mammals, including those related to sex drive, mate choice, and attachment (Fisher, 1998, 2000; Fisher et al., 2002; Broad et al., 2006; Carter and Perkeybile, 2018; Curley and Keverne, 2005; Fisher et al., 2006; Fischer et al., 2019; Johnson and Young, 2015; Numan and Young, 2016; Porges, 1998). Romantic love may have evolved after the neural circuitry associated with mate choice became populated by oxytocin receptors which played a role in the evolution of enduring social attraction and pair-bond formation (see Numan and Young, 2016). “[P]air bonding is the evolutionary antecedent of romantic love and…the pair bond is an essential element of romantic love” (Walum and Young, 2018, p. 12).

**FIGURE 1:**
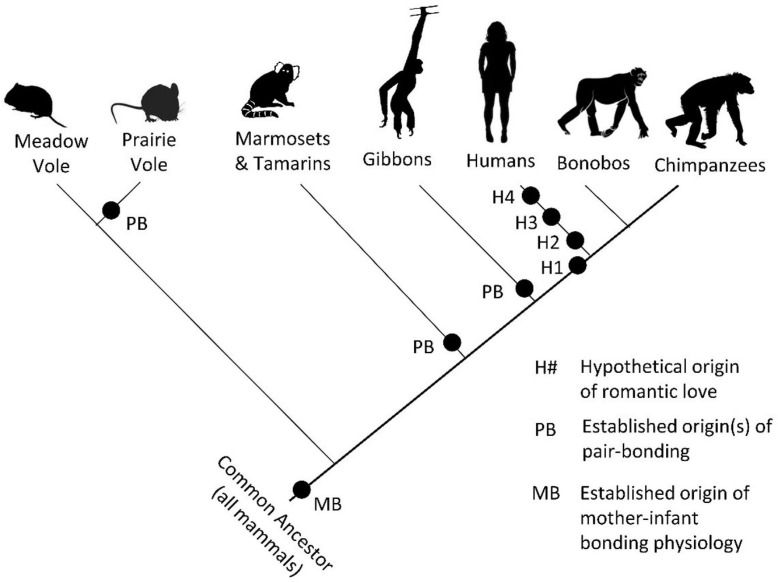
Phylogenetic relationships among select mammal species that pair-bond.

Examining the similarities between the neurobiological and endocrinological mechanisms involved in mother-infant bonding and pair-bonding in mammals, it becomes apparent that the maternal functions of this suite of adaptations arose deep in the evolutionary history of mammals (Numan and Young, 2016). Their derived, pair-bonding functions would have arisen later in a very small number of species (only 3–5% pair-bond). As such, the neural circuitry and other proximate mechanisms involved in mother-infant bonding in mammals “may have provided a primordial neural scaffold upon which other types of strong social bonds, such as pair bonds, have been built” (Numan and Young, 2016, p. 99). We are, thus, on reasonably solid ground to posit evolutionary trajectories of romantic love. Figure 1 presents information and hypotheses about the evolutionary history of romantic love. Evolutionary trajectories of romantic love start with the ancestral mammalian mother–infant bonding mechanisms and culminate in their co-option and modification for pair-bonding in several mammalian lineages (Numan and Young, 2016). Human romantic love results from one of these trajectories. In another trajectory—the one that includes pair-bonding prairie voles (*Microtus ochrogaster*)—we know quite a lot about the functioning of oxytocin, vasopressin, and dopamine in facilitating pair-bonding (e.g., Carter and Getz, 1993; Carter and Perkeybile, 2018; Walum and Young, 2018). Although these derived changes to the primitive mammalian machinery may not be the direct evolutionary antecedents of those at work in humans (they are, rather, the product of parallel evolution), they provide a window into how basic machinery can be modified to affect those ends. One substantive difference appears to be the relative importance of the hormonal drivers in the smaller species, and the dopamine-related ones in humans (Broad et al., 2006; Fisher et al., 2016).

#### Pair-Bonds in Primates

Humans are members of the primate superfamily Anthropoidea, amongst whom there is great diversity in social systems, and whose ancestral state likely included complex group-based social relationships (Kay et al., 1997; Shultz and Dunbar, 2007). This would have included long-term association between unrelated males and females—which is a far cry from the solitary system that is modal and ancestral for mammals (Lukas and Clutton-Brock, 2013; Opie et al., 2013). There are even some members of this lineage who have evolved pair-bonds, such as the marmosets and tamarins (Callitrichidae), and gibbons (Hylobatidae). The similarities between these species and humans in terms of the adaptive suite related to pair-bonds, like the similarities between humans and voles, are due to convergent/parallel evolution (French et al., 2018).

Our closest living relatives are the common chimpanzee (*Pan troglodytes*) and bonobo (*Pan paniscus*) with whom we share a common ancestor just 5–8 million years ago. While bonobos are alluring due to their free-willed sexual nature, common chimpanzees provide a better glimpse into the behavior of our direct ancestors. Although the common chimpanzee mating system is defined as promiscuous, there are, in fact, three forms of common chimpanzee mating tactics (Morin, 1993). The first two—possessive mating and consortships—involve some of the characteristics we associate with romantic love, such as a more-than-fleeting association and mate guarding, but they are much rarer than the third type, opportunistic mating. The comparison of chimpanzees and humans, thus, suggests that one possible hypothesis for the emergence of romantic love is that it originated in their common ancestor (H1 in Figure 1). Alternatively, it might be that the common ancestor had an adaptive repertoire that was primed for its emergence when the requisite socioecological context arose. In this way, the evolution of romantic love from chimp-like mating is similar to the evolution of human culture from chimp-like culture.

For some, the origin of romantic love was more likely to have fallen somewhere on our side of the human–chimpanzee split (e.g., Fisher et al., 2016). Even so, we are left with the difficulty of pinpointing exactly when it arose—attributable to there being only one extant hominin species from amongst the many that have existed (Pigliucci and Kaplan, 2006) and the lack of direct fossil evidence for romantic love. If we accept the conventional view that romantic love evolved to facilitate pair-bonding, then we can search for clues about the evolution of the former by tracing the evolution of the latter (Fletcher et al., 2015). A transition from ape-like to human-like sexual behavior in our lineage may have pre-dated the emergence of the genus *Homo* (Lovejoy, 1981)—and, thus, we have a second hypothesis (H2 in Figure 1). A comparison of sexual dimorphism in *Australopithecus* and early genus *Homo*, however, suggests a third hypothesis—that it arose after their emergence (H3 in Figure 1). Several lines of evidence suggest that the earliest members of our species, *Homo sapiens*, pair-bonded but were not necessarily monogamous. Based on an examination of the distribution of mating systems in modern, small-scale human societies and three correlates of primate mating systems (Dixson, 2009), it is possible to conclude that pair-bonds are a “ubiquitous” feature of human mating that can manifest through polygyny or polyandry, but most commonly occur in the form of serial monogamy (Schacht and Kramer, 2019). The final hypothesis, thus, is that romantic love is the unique domain of our species (H4 in Figure 1).

The transition to mostly monogamous and some polygynous groupings could have had a transitional phase where polygynous groupings were the norm (Chapais, 2008, 2013). Pair-bonds may have arisen from a complex interaction between the fitness benefits and costs of mating and parental care (Quinlan, 2008). The transition from ape-like promiscuity to human pair-bonds may have been driven by the provision of females by low-ranking males (Gavrilets, 2012). The direct benefits for females was the food provided, for the males, the mating opportunity. This may have led to selection for males that were less aggressive and more prosocial. The female mate-choice mechanism is a distinct possibility for explaining human self-domestication (Gleeson and Kushnick, 2018).

#### Gene-Culture Coevolution

Romantic love is a universal or near-universal feature in human societies (Jankowiak and Fischer, 1992; Gottschall and Nordlund, 2006; Jankowiak and Paladino, 2008; Fletcher et al., 2015; Buss, 2019; Sorokowski et al., 2020). A small number of genetic correlation studies show that there are a number of genes associated with romantic love (Emanuele et al., 2007; Murray et al., 2019; Acevedo et al., 2020). Other insights into the genetic evolution of romantic love can be garnered from elsewhere, however. For example, life history theory provides insight into ethnic or geographical variation in romantic love and its role in providing sexual access by women.

Romantic love is among the most common reasons female adolescents give for having sex (Ozer et al., 2003). A “slow” life history strategy is associated with eros more than other loving styles (Marzec and Łukasik, 2017). Psychopathology associated with impulsivity is a feature of a “fast” life history strategy, as is promiscuous sexuality (Del Giudice, 2016). Greater impulsivity is associated with a reduced likelihood of giving romantic love as a reason for having sex among adolescent females (Dawson et al., 2008).

As a result, genetic determinants of life history strategies (e.g., Figueredo et al., 2004) may influence the occurrence of romantic love. National scores on the life history strategy genetic factor index correlate with adolescent fertility rates indicating that genetic predictors of a fast life history are associated with higher rates of adolescent pregnancy (Luoto, 2019b). This ethnic or geographical variation in the genetic determinants of life history strategies may also represent ethnic or geographic variation in the genetic determinants and reproductive relevance of romantic love.

In addition to this, cultural factors may have affected the role of romantic love in mating and marriage decisions—and this has implications for understanding the evolution of romantic love (Fletcher et al., 2015). Arranged marriages are the norm in 80% of 200 forager societies from the Ethnographic Atlas (Apostolou, 2007). Phylogenetic methods to reconstruct the ancestral marriage patterns of our species using the same data found that there were likely marriage transactions (brideprice or brideservice) but only a limited amount of polygyny (Walker et al., 2011). While the ancestral state for arranged marriages was not definitive, arranged marriages were likely present around 50 thousand years ago, when our ancestors expanded their range beyond Africa. So, despite romantic love being viewed as an important component of marriage and mating, it may have played a role of decreasing importance in the recent evolutionary history of our species. Arranged marriages may have limited the role of female mate choice in intersexual selection (Apostolou, 2007). Further, despite romantic love’s decreased role in courtship and marriage, it may have continued to serve a role in facilitating pair-bonding as romantic love can develop even in the arranged-marriage context. The role of romantic love in facilitating mate choice, courtship, and marriage may now be increasing with the decline and modification of arranged marriages in many parts of the world (e.g., Allendorf and Pandian, 2016). This may be the result of the increasing sexual equality of women (e.g., de Munck and Korotayev, 1999).

## Discussion

Romantic love is a complex and multifaceted aspect of human biology and psychology. Our approach in this review has been to highlight how Tinbergen’s (1963) “four questions” can help us to synthesize the important strands related to the mechanisms, development, fitness-relevant functions, and evolutionary history of this phenomenon. Here, we synthesize what this review has presented in each level of explanation and suggest what this indicates about other levels of explanation. We then highlight some gaps in our knowledge that could be filled with future research and present a new ethologically informed working definition of romantic love.

### What Do Tinbergen’s Four Questions Tell Us?

One of the benefits of using Tinbergen’s four questions as a framework to describe a complex trait such as romantic love is its ability for one level of explanation to provide insights into the other level of explanation (see Tinbergen, 1963; Bateson and Laland, 2013; Zietsch et al., 2020). In particular, an understanding of the proximate causes of romantic love has provided insights into the functions and phylogeny of romantic love although an understanding of the ultimate level of explanation provides some insights into the mechanisms of romantic love.

Multiple mechanistic systems involved in romantic love suggests it may serve multiple functions and may be a suite of adaptations and by-products rather than a single adaptation. We found that romantic love is associated with activity in a number of neural systems: reward and motivation, emotions, sexual desire and arousal, and social cognition. It is also associated with activity in higher-order cortical brain areas that are involved in attention, memory, mental associations, and self-representation. We also found that romantic love is associated with a number of endocrine systems: sex hormones, serotonin, dopamine, oxytocin, cortisol, and nerve growth factor. This is consistent with our position that romantic love serves mate choice, courtship, sex, and pair-bonding functions. Reward and motivation system activity may be particularly involved in the mate choice function of romantic love. Cortisol may be particularly indicative of the courtship function of romantic love, which overlaps with pair-bonding. Neural areas associated with sexual desire and arousal and the activity of sex hormones may play a particular role in the sex function. Finally, reward and motivation regions of the brain (rich with oxytocin receptors) and activity of the oxytocin system may play a particular role in the pair-bonding function of romantic love. Our understanding of the biological mechanisms that cause romantic love supports our description of romantic love’s functions.

Mechanistic similarities between romantic love and mother-infant bonding suggest that romantic love may have evolved by co-opting mother-infant bonding mechanisms. This articulates one hypothesis about the evolutionary history of romantic love that complements the predominate theory of independent emotion systems (Fisher, 1998, 2000; Fisher et al., 2002). This is supported by the psychological similarities between romantic love and early parental love.

Evidence of substantial activity of oxytocin receptor rich brain regions and the oxytocin endocrine system in romantic love lends weight to the position that romantic love only evolved after the neural circuitry associated with mate choice, specifically, regions of the mesolimbic reward pathway and dopamine rich areas, became populated by oxytocin receptors specifically receptive to stimuli from mating partners. That played a role in the evolution of enduring social attraction and pair-bond formation (Numan and Young, 2016). This supports our claim that romantic love probably evolved in conjunction with pair-bonds in humans. As a result, we are bolstered when we contend that romantic love emerged relatively recently in the history of humans.

The duration of romantic love also raises questions about the functions of romantic love. It has been said that the psychological features of romantic love can last from 18 months to 3 years in reciprocated romantic love. However, in our evolutionary history, romantic love would have usually occurred in the context of pregnancy and child birth. Mother-infant bonding becomes active around the time of childbirth. We are not aware of any research that has investigated whether romantic love can occur at the same time as mother-infant bonding or whether it must subside for mother-infant bonding to become active. Answering this question would elucidate if the functions of romantic love extinguish once reproduction has been successful. The existence of long-term romantic love also raises questions about the functions of romantic love. It has been posited that long-term romantic love is “part of a broad mammalian strategy for reproduction and long-term attachment” (Acevedo et al., 2020, p. 1). This may indicate that long-term romantic love serves similar functions to romantic love that lasts a shorter period of time.

Just as the multiple biological mechanisms involved in romantic love suggests a variety of functions, the functions of romantic love specified in our review suggests specific biological mechanisms are involved. As outlined above, specific functions may be associated with specific mechanisms and this should be an area of targeted research.

The possibility of romantic love evolving by co-opting mother-infant bonding mechanisms raises a number of possibilities in relation to the proximate causes of romantic love. It suggests that social activity associated with mother–infant bonding (e.g., filling of needs, specific cues) may be particularly important precursors to, or features of, romantic love. It suggests that many of the genes and polymorphisms involved in causing romantic love may have been present in mammals since the emergence of mother–infant bonding, making comparative animal research using mammals relevant. It also suggests that further research into shared neural activity between romantic love and mother–infant bonding is warranted.

We contend that romantic love probably emerged in conjunction with pair-bonds in humans or human ancestors. As such, further information about the similarities and differences between romantic love (pair-bonding) and companionate love (established pair-bonds) is needed. In particular, information about any role of the mesolimbic pathway (see Loth and Donaldson, 2021) or regions associated with sexual desire in companionate love would help to shed light on the evolutionary history of pair-bonding and pair-bonds. Specifically, this could shed light on if, as has been suggested (see Walum and Young, 2018), romantic love and pair-bonds are inextricably linked.

### Areas of Future Research

One issue with research into the mechanisms of romantic love is that it has, with some exceptions (e.g., Fisher et al., 2010), utilized samples of people experiencing romantic love who are in a relationship with their loved one. Romantic love serves a mate choice and courtship function, and as a result, a large proportion of people experiencing romantic love are not in a relationship with their loved one (e.g., Bringle et al., 2013). A small number of studies have directly investigated unrequited love (e.g., Tennov, 1979; Baumeister et al., 1993; Hill et al., 1997; Aron et al., 1998; Bringle et al., 2013), but none of these investigated the mechanisms that cause romantic love. Studying such people might identify the specific contributions of particular mechanisms to particular functions. For example, the mechanisms associated with the pair-bonding function of romantic love may not be active in individuals who are engaging in courtship and the mechanisms involved in courtship may not be present in lovers who are already in a relationship with their loved one. Research would benefit from considering the mechanisms that underlie related psychopathologies and it would be useful to understand the relationship between mate preferences and romantic love.

Molecular genetics research, such as that undertaken by Acevedo et al. (2020), could further identify contributions of genes in people experiencing romantic love. Resting state fMRI provide an opportunity to investigate networks characteristic of psychopathology related to romantic love. Research should investigate the automatic/internal emotional regulatory network and the volitional/external regulatory network associated with mania/hypomania in people experiencing romantic love. Further research is required into the endocrinology of romantic love. In particular, further research is needed into the role of opioids, corticotropin-releasing factor, glutamate, acetylcholine, and vasopressin in romantic love. Efforts should be made to combine psychological and mechanisms research. For example, differences in neural or endocrine activity may be present in people experiencing romantic love who display elevated symptoms of depression compared to those who display reduced symptoms. As a result, neuroimaging and endocrinological studies could categorize people experiencing romantic love according to their levels of depression or type of hypomanic symptoms.

Given the large number of fMRI studies, interpreting the neuroimaging literature can be overwhelming. It has been nearly 10 years since the last meta-analysis of fMRI studies including romantic love. It is time for another one that focuses solely on romantic love. There is also a pressing need to attempt to replicate and extend endocrine studies and to specifically investigate the oxytocin system in people experiencing romantic love using validated measures of romantic love. As with many areas of psychological research (Henrich et al., 2010), and specifically in areas related to mating psychology (Apicella et al., 2019; Scelza et al., 2020), there is a pressing need to ensure that samples used in research are not exclusively Western, educated, industrialized, rich, and democratic.

Limited ontogeny research has elucidated the mechanisms causing romantic love across the lifespan. The literature that has (e.g., Luoto, 2019a), has focused on mate choice, rather than romantic love, *per se*. We know nothing about the neurobiology or endocrinology of romantic love in children or about the endocrinology of long-term romantic love. It would be useful to investigate how the functions of romantic love differ according to age of individuals or the duration of romantic love. Internal and external factors influence romantic love, although there has been surprisingly little research into this topic. It would be prudent to continue to develop a more detailed understanding of the factors that lead to romantic love (e.g., Riela et al., 2010, 2017). It would be useful to better understand the relationship between attachment styles and romantic love. Research should investigate if romantic love can occur at the same time as mother-infant bonding, or if they are mutually exclusive states.

Research into the functions of romantic love is sparse. There is a need for clear, evidence-informed definitions and descriptions of each of the functions of romantic love. It is likely that different mechanisms moderate different functions, and research should attempt to determine the contribution of specific genetic, neural, and endocrine activity to each individual function (see Zietsch et al., 2020). The advent of contraception and the adoption of family planning strategies means romantic love now serves more of a sex function than a pregnancy function in some environments. This is particularly the case early in a relationship. Pregnancy may become a feature as a relationship progresses and the fitness consequences of romantic love need to be investigated. Romantic love’s role as a suite of adaptations and by-products should be investigated. There is theoretical support for the notion that romantic love serves a health-promoting function (e.g., Esch and Stefano, 2005); however, there is a limited number of studies demonstrating a health-promoting effect of romantic love.

The relative infancy of genetic research, the lack of a clear fossil record, and the small number of species with which comparative analysis can be undertaken, means novel and creative means of investigating the phylogeny of romantic love must be undertaken. There is a need to pin-point the phylogenetic emergence of romantic love and the factors that caused it. To do this, more research into the genetics of romantic love must be conducted, and this should consider the phylogeny of specific genes and polymorphisms (e.g., Acevedo et al., 2020; see also Walum and Young, 2018). Efforts to assess the contribution of sexual selection to the evolution of romantic love are warranted. Studies of newly discovered fossils can help to identify shifts in sexual dimorphism that are indicative of pair-bonds. Further observational and experimental research into romantic love in hunter-gatherer tribes could tell us more about how romantic love functioned in our evolutionary history. Comparative research still has much to contribute. Research should explore the possibility that initial changes to the ancestral mammalian physiology that led directly to human romantic love arose in response to selection on both mating and non-mating-related behavior, such as pro-sociality (e.g., Barron and Hare, 2020; Luoto, 2020) or unique aspects of our species’ parenting repertoire. It might be fruitful to further investigate the relationship between romantic love and life history theory (e.g., Olderbak and Figueredo, 2009; Marzec and Łukasik, 2017). Finally, efforts should be made to elaborate and test the theory that romantic love emerged by co-opting mother–infant bonding mechanisms.

### A New Working Definition of Romantic Love

The introduction to this review provided four definitions or descriptions of romantic love. For decades, most definitions (Hendrick and Hendrick, 1986; Sternberg, 1986; Hatfield and Rapson, 1993) of romantic love have informed research into the cognitive, emotional, and behavioral characteristics of romantic love. The past two decades, however, have seen an increasing focus on the biology of romantic love. Only recently has an evolution-informed definition been proposed (Fletcher et al., 2015). That working definition, however, does not incorporate much of the research that provides insight into the proximate and ultimate causes of romantic love.

We believe that the analytical approach taken in this review has identified sufficient information to justify the development of a new ethologically informed working definition of romantic love. The purpose would be to create an inclusive definition that is useful for researchers in varied disciplines investigating romantic love’s psychological characteristics, genetics, neurobiology, endocrinology, development, fitness-relevant functions, and evolutionary history. It may also be of use to psychologists and psychiatrists attempting to understand the experience and etiology of romantic love in their practice. It should be sufficiently precise and descriptive to both guide and link research. We provide, here, a working definition of romantic love:

Romantic love is a motivational state typically associated with a desire for long-term mating with a particular individual. It occurs across the lifespan and is associated with distinctive cognitive, emotional, behavioral, social, genetic, neural, and endocrine activity in both sexes. Throughout much of the life course, it serves mate choice, courtship, sex, and pair-bonding functions. It is a suite of adaptations and by-products that arose sometime during the recent evolutionary history of humans.

We situate the study of romantic love within the context of existing human mating literature. Our definition recognizes that romantic love is experienced across the lifetime of an individual, that research has shed light on the social, psychological, genetic, neural, and endocrine characteristics associated with it, and that it occurs in both sexes. Our definition also recognizes that romantic love serves a variety of functions and that these functions may vary across the lifespan. It does not exclude long-term or unrequited romantic love from the definition. Health is not identified as a function of romantic love in our definition despite being considered in our review. If more evidence comes to light, this definition can be amended to incorporate health.

Our definition has similarities and differences with the definition proposed by Fletcher et al. (2015). This is appropriate given both are informed by evolutionary approaches which differ somewhat. We do not specifically define romantic love as being a commitment device or reference passion, intimacy, and caregiving. In our review, we recognize that romantic love is a commitment device and serves to display commitment and signal fidelity as part of its courtship function. We believe that reference to romantic love’s behavioral activity and courtship and pair-bonding functions sufficiently encapsulate this concept. Sternberg’s (1997) definition of romantic love and Fletcher et al.’s (2015) definition include references to passion and intimacy. Caregiving (e.g., provision of psychological and emotional resources, sharing resources), while associated with pair-bonding, is not sufficiently definitive of romantic love using Tinbergen’s four questions as a framework to include in our definition.

We do not reference the universality of romantic love. While some experts assert its universality (e.g., Fletcher et al., 2015; Buss, 2019), we believe that the finding of Jankowiak and Fischer (1992) leaves enough uncertainty for it to be prudent to omit this aspect from our definition. Their research has found no evidence of romantic love in fifteen cultures (see Jankowiak and Paladino, 2008, for update to the original investigation) although this is probably the result of lack of data rather than evidence to the contrary. Once this matter is settled, which could be achieved by further investigating those societies where no evidence of romantic love was found, the definition can be amended. Fletcher et al. (2015) state that romantic love is associated with pair-bonds. We do the same by stating that pair-bonding is one of the functions of romantic love.

We also do not make specific reference to romantic love suppressing the search for mates. We recognize this as a cost in our review, but do not believe that this is so definitive of romantic love to include in our definition. Rather, we believe that our reference to “behavioral” activity and the “mate choice” function of romantic love in our definition sufficiently accommodates this feature. Our definition provides more detail than that provided by Fletcher et al. (2015) by including elements derived from substantial research into the mechanisms, ontogeny, functions, and phylogeny of romantic love. Like the Fletcher et al. (2015) definition, our definition recognizes that romantic love has distinct psychological characteristics and that we know about some of the proximate mechanisms that regulate it. However, as explained above, we do not include reference to the health-promoting effects of romantic love.

As more information about romantic love is gathered, we anticipate the definition to develop. However, we believe that this definition is an improvement upon previous definitions and adequately captures what is currently known about romantic love’s proximate and ultimate causes. It would be useful for researchers investigating romantic love from myriad perspectives. This definition should be critiqued and improved, and we welcome any such efforts from researchers and theorists across the spectrum of academic disciplines.

## Conclusion

Our review provides a comprehensive account of the phenomenon known as romantic love. It covers topics such as social precipitants, psychology, genetics, neurobiology, and endocrinology. It provides an account of romantic love across the lifetime of an individual and is the first to propose four discrete reproduction-related functions of romantic love supported in the literature: mate choice, courtship, sex, and pair-bonding. It provides a summary of the benefits and costs of romantic love, outlines possible selective pressures, and posits that it is a complex suite of adaptations and by-products. We propose four potential evolutionary histories of romantic love and introduce the theory of co-opting mother-infant bonding mechanisms. We have identified a number of specific and general areas for future research. Our review suggests a new, ethologically informed working definition of romantic love that synthesizes a broad range of research. The working definition we propose serves to define a complex trait in a way that can both guide and link research from a variety of fields.

## Author Contributions

AB conceived the manuscript. AB and GK collaborated on the development of the analytical framework and writing of the manuscript. Both authors approved the final version.

## Conflict of Interest

The authors declare that the research was conducted in the absence of any commercial or financial relationships that could be construed as a potential conflict of interest.
