# Corrigendum: Proximate and Ultimate Perspectives on Romantic Love

**DOI:** 10.3389/fpsyg.2021.694913

**Published:** 2021-06-24

**Authors:** Adam Bode, Geoff Kushnick

**Affiliations:** Human Behavioural Ecology Research Group, School of Archaeology and Anthropology, ANU College of Arts and Social Sciences, The Australian National University, Canberra, ACT, Australia

**Keywords:** romantic love, mechanisms, ontogeny, functions, phylogeny, Tinbergen, human mating, definition

In the original article, there was an error. In the introduction we state “Romantic love serves a variety of functions that vary according to life-stage and duration, including mate choice, courtship, pregnancy, and pair-bonding.” In this sentence, “pregnancy” should be “sex.”

A correction has been made to *Introduction, Paragraph 1*. The corrected paragraph is shown below.

Romantic love is a complex suite of adaptations and by-products that serves a range of functions related to reproduction (Fletcher et al., 2015; Buss, 2019). It often occurs early in a romantic relationship but can lead to long-term mating. It is a universal or near-universal (Jankowiak and Fischer, 1992; Gottschall and Nordlund, 2006; Jankowiak and Paladino, 2008; Fletcher et al., 2015; Buss, 2019; Sorokowski et al., 2020) and is characterized by a range of cognitive, emotional, behavioral, social, genetic, neural, and endocrine activity. It occurs across the lifespan in both sexes. Romantic love serves a variety of functions that vary according to life-stage and duration, including mate choice, courtship, sex, and pair-bonding. Its evolutionary history is probably coupled with the emergence of pair-bonds relatively recently in human evolutionary history.

Additionally, in the original article, there was an error in the subsection “Sex”. Here, we stated that “The costs associated with romantic love's reproduction function are far greater for women than for men (Trivers, 1972).” In that sentence, the word “reproduction” should be “sex.”

A correction has been made to *Ultimate Perspectives, Sex, Paragraph 5*. The corrected paragraph is shown below.

The costs associated with romantic love's sex function are far greater for women than for men (Trivers, 1972). Both sexes could be subject to unwanted pregnancy and associated parenting responsibilities (although this impacts women to a greater extent). There is also, however, a risk of damage to an individual's reputation. Women are often subject to criticism from other women for engaging in sexual activity (Koehn and Jonason, 2018), especially if a long-term relationship does not result. Men and women risk damage to their reputation for having sex with a low mate value partner, although men are generally treated far more favorably than women for engaging in sexual activity (see Zaikman and Marks, 2017). For women, a period of pregnancy followed by a lengthy period of lactation may ensue, and this is costly in terms of the ability to obtain sufficient resources and protecting oneself from harm. There is also the possibility that the relationship will dissolve following pregnancy and the woman may be left to raise a child without the father's support (Koehn and Jonason, 2018).

The authors apologize for these errors and state that they do not change the scientific conclusions of the article in any way. The original article has been updated.
