# Supplementary material for: Proximate and Ultimate Perspectives on Romantic Love
Source: Front Psychol. 2021 Apr 12;12:573123. doi: 10.3389/fpsyg.2021.573123 (PMC8074860; doi:10.3389/fpsyg.2021.573123)
Supplement: Supplementary file 1 [file Table_1.docx]

| **Table S1. Studies** **investigating hypomania, depression, and anxiety in people experiencing romantic love** | | | | |
| --- | --- | --- | --- | --- |
| **Authors** | **Sample** | **Measure of romantic love** | **Symptoms investigated** | **Measures used** |
| Hatfield, Brinton, & Cornelius (1989) | Study 1: n=41 (f=17); F age=12.5; M age=12.3  Study 2: n=64 (f=32); Age=14.5 | JLS  JLS | Trait anxiety  State-trait anxiety | CAS  STAI-C |
| Wang (1994) | Americans: n=57 (f=31)  Italian: n-55 (f=25) | PLS | Social anxiety | SAD |
| Wang & Nguyen (1995) | Adolescents: n=89 (f=51); F age=15.01 (SD=.66); M age=15.48 (SD)=.55  Young adults: n=62 (f=33); F age=25.97 (SD=2.39); M age=26.03 (SD=2.86)  Middle aged adults: n=51 (f=30); F age=40.21 (SD=5.70); M age=38.67 (SD=6.67)  Elderly adults: n=53 (f=35); F age=67.25 (SD6.53); M age=65.47 (SD=8.6) | PLS | State-trait anxiety | STAI |
| Brand, Luethi, von Planta, Hatzinger, & Holsboer-Trachsler (2007) | n=113 (f=72); F age=17.81 (SD=1.39); M age=18.19 (SD=1.21);  Romantic love sample n=65 (f=40; m=25) | Self-report “recently fallen in love experiencing intense romantic love”; 3 adapted questions from the Y-BOCS on a five-point scale. | Hypomania | HCL-32 |
| Bajoghli, Jashaghani, Mohammadi, Holsboer-Trachsler, & Brand (2011) | n=86 (f=86); Age=17.97 (SD=1.09)  Romantic love sample n=45; Mean relationship duration=10.10 months; SD=5.84 months) | Self-report “Are you actually in love with another person?” | Hypomania; Depression | HCL-32; VZDS |
| Bajoghli et al. (2013) | n=201 (f=113; m=88); Age=17.73 (SD=1.06);  Romantic love sample n=81 (f=48; m=33); Mean relationship duration=10.66 months (SD=7.4) | Self-report “Are you currently in love with another person?” | Hypomania; depression; State-trait-anxiety | HCL-32; VZDS; STAI |
| Bajoghli et al. (2014) | n=100 (f=47; m=53); F age=28.79 (SD=2.38); M age=26.11 (SD=2.63);  Romantic love sample n=96 | Self-report “Are you currently in love with another person?”; 3 adapted questions from the Y-BOCS on a five-point scale. | Hypomania; depression; State-trait anxiety | HCL-32; BDI; STAI |
| Brand et al. (2015) | n=844 (f=640; m=204); F age=24.47 (SD=5.23); M age=25.79 (SD=6.49) | Self-report “Are you currently in love with another person?”; 3 adapted questions from the Y-BOCS on a five-point scale. | Hypomania; Depression, State-trait anxiety | HCL-32; BDI; STAI |
| Bajoghli et al. (2017) | n=157 (f=90; m=67); Age=18.97 (SD=1.34); Follow up from (Bajoghli et al., 2013) study | Self-report “Are you currently in love with another person?” | Hypomania; Depression; State-trait anxiety | HCL-32; VZDS; STAI |
| Kuula, Partonen, & Pesonen (2020) | n=1374 (f=913); Age=16.8 (SD=.58) | Self-report “Love can be a powerful feeling, especially in the beginning. Are you currently experiencing the emotional turmoil which relates to the early stages of falling in love?” | Anxiety; Depression | GAD-7; BDI-II |
| F=female; M=male; JLS= Juvenile Love Scale; CAS=Child Anxiety Scale; STAI-C= State-Trait Anxiety Inventory for children; SAD=Social Avoidance and Distress Scale STAI= State-Trait Anxiety Inventory; PLS=Passionate Love Scale; Y-BOCS=Yale-Brown Obsessive Compulsive Scale; HCL-32=Hypomanic Check List-32; VZDS= Von Zerssen’s Depression Scale; BDI=Beck Depression Inventory; BDI-II= Beck Depression Inventory-II; GAD-7= Generalized Anxiety Disorder-7 | | | | |

**References**

Bajoghli, H., Farnia, V., Joshaghani, N., Haghighi, M., Jahangard, L., Ahmadpanah, M., . . . Brand, S. (2017). "I love you forever (more or less)'' - stability and change in adolescents' romantic love status and associations with mood states. *Revista Brasileira De Psiquiatria, 39*(4), 323-329. doi:10.1590/1516-4446-2016-2126

Bajoghli, H., Joshaghani, N., Gerber, M., Mohammadi, M. R., Holsboer-Trachsler, E., & Brand, S. (2013). In Iranian female and male adolescents, romantic love is related to hypomania and low depressive symptoms, but also to higher state anxiety. *International Journal of Psychiatry in Clinical Practice, 17*(2), 98-109. doi:10.3109/13651501.2012.697564

Bajoghli, H., Joshaghani, N., Mohammadi, M. R., Holsboer-Trachsler, E., & Brand, S. (2011). In female adolescents, romantic love is related to hypomanic-like stages and increased physical activity, but not to sleep or depressive symptoms. *International Journal of Psychiatry in Clinical Practice, 15*(3), 164-170. doi:10.3109/13651501.2010.549340

Bajoghli, H., Keshavarzi, Z., Mohammadi, M. R., Schmidt, N. B., Norton, P. J., Holsboer-Trachsler, E., & Brand, S. (2014). "I love you more than I can stand!" - Romantic love, symptoms of depression and anxiety, and sleep complaints are related among young adults. *International Journal of Psychiatry in Clinical Practice, 18*(3), 169-174. doi:10.3109/13651501.2014.902072

Brand, S., Foell, S., Bajoghli, H., Keshavarzi, Z., Kalak, N., Gerber, M., . . . Holsboer-Trachsler, E. (2015). "Tell me, how bright your hypomania is, and I tell you, if you are happily in love!"-Among young adults in love, bright side hypomania is related to reduced depression and anxiety, and better sleep quality. *International Journal of Psychiatry in Clinical Practice, 19*(1), 24-31. doi:10.3109/13651501.2014.968588

Brand, S., Luethi, M., von Planta, A., Hatzinger, M., & Holsboer-Trachsler, E. (2007). Romantic love, hypomania, and sleep pattern in adolescents. *Journal of Adolescent Health, 41*(1), 69-76. doi:10.1016/j.jadohealth.2007.01.012

Hatfield, E., Brinton, C., & Cornelius, J. (1989). Passioante love and anxiety in young adolescents. *Motivation and Emotion, 13*(4), 271-289. doi:10.1007/bf00995539

Kuula, L., Partonen, T., & Pesonen, A. K. (2020). Emotions relating to romantic love-further disruptors of adolescent sleep. *Sleep Health, 6*(2), 159-165. doi:10.1016/j.sleh.2020.01.006

Wang, A. Y. (1994). Passionate love and social anxiety of American and Italian students. *Psychology, 31*(3-4), 9-11. Retrieved from <Go to ISI>://WOS:A1994QB92000003

Wang, A. Y., & Nguyen, H. T. (1995). Passionate love and anxiety - A cross-generational study. *Journal of Social Psychology, 135*(4), 459-470. doi:10.1080/00224545.1995.9712215
