# Supplementary material for: Proximate and Ultimate Perspectives on Romantic Love
Source: Front Psychol. 2021 Apr 12;12:573123. doi: 10.3389/fpsyg.2021.573123 (PMC8074860; doi:10.3389/fpsyg.2021.573123)
Supplement: Supplementary file 2 [file Table_2.docx]

| **Table S2: fMRI studies investigating romantic love** | | | | |
| --- | --- | --- | --- | --- |
| **Study** | **Romantic Love Sample** | **Measure of romantic love** | **Control/ comparison sample** | **Romantic love stimulus** |
| Bartels & Zeki (2000) | n=17 (f=11); Mean age=24.5; Mean duration of relationship=2.4 years (SD=1.7 years) | Short written statement about how much they were in love; Interview; PLS (Short version) mean items score=7.55; SD=0.97) | nil | Photo of loved one |
| Bartels & Zeki (2004) | See Bartels & Zeki (2000) | See Bartels & Zeki (2000) | n=20 (f=20); Mothers; Mean age=34; Children mean age=24.4 months (SD=15.7) | See Bartels & Zeki (2000) |
| Aron et al. (2005) | n=17 (f=10); Mean age=20.6; | Semi-structured interview; PLS mean item score=8.54; Mean in love duration=7.4 months | nil | Photo; Pleasurable but not sexual thoughts of loved one (30 seconds) |
| Ortigue, Bianchi-Demicheli, Hamilton, & Grafton (2007) | n=36 (f=36); Mean age 20.1 (SD=3.2) | Semi-structured interview; Intensity of love; Percentage of time thinking about beloved in waking hours; PLS mean item score=7.7 (SD=7.85); Mean duration of love=15.3months (SD=14.5) | nil | Name (prime; 26 msec) |
| Kim et al. (2009) | n=10 (f=5); Mean age 21.1 (SD=1.97); Dating less than 100 days | PLS Baseline mean f=120.2; SD=7; Baseline mean m=118.6; SD=9.1; Follow-up f=106.2; SD 3.0; Follow-up mean m=110.8; SD=4.0 | nil | Photos depicting various facial expressions of the loved one (30 seconds) |
| Fisher, Brown, Aron, Strong, & Mashek (2010) | n=10 (f=10); Mean age 19.8 (SD=1; Mean duration of relationship before breakup=21months; Mean time since rejection=63 days | PLS mean items score=8 (SD=0.6); Percentage of day spent thinking about sweetheart (mean=85%) | nil | Photo; Thinking about events that occurred with the rejecter |
| Younger, Aron, Parke, Chatterjee, & Mackey (2010) | n=15 (f=8); Mean age=20: Relationship duration ≥9 months | PLS (short version) mean score=109.8 (SD=11.2) | nil | Photo, focus and think about loved one |
| Zeki & Romaya (2010) | n=28 (4 excluded) (f=14); Mean age=26.3 (SD=6.4); Mean relationship duration=3.7 months (SD=4.4) | PLS mean score=100.1 | Heterosexual (n=12) v homosexual (n=12) | Photo (16 seconds) |
| Xu et al., (2011) | n=18 (f-10); Mean age=261.61 (SD=1.75); In a relationship | nil | nil | Photo; Pleasurable but not sexual thoughts of loved one (30 seconds) |
| Stoessel et al. (2011) | n=12 (f=6); Mean age=24.2 (SD=4.02); PLS score >85 | In love for less than 6 months; PLS mean f scores=112.4 (SD=14.76); Mean m score=109.8 (SD=12.69); | n=12 (f=6); Mean age=24.08 (SD=4.5); Beck Depression Inventory mean m score=21.0 (SD=11.98); Mean f score=23.0 (SD=8.60) | Photo (12 seconds)  Text (12 seconds) |
| Acevedo, Aron, Fisher, & Brown (2012) | n=17 (f=10); Mean age=52.85 (SD=8.91); >10 years relationship length; Mean marriage duration=21.4 years (SD=5.89) | PLS mean item score=5.51 (SD=0.36); Eros subscale of the LAS mean item score=5.76 (SD=0.26) | nil | Photo; Pleasurable but not sexual thoughts of loved one (30 seconds) |
| Xu et al. (2012a) | n=18 (1 excluded) (f=0); Mean age=25.11 (SD=3.03); Mean cigarettes per day=15.78 (SD=7.83); Mean smoking duration=4.42 years (SD=2.70 years); Non-smoking romantic partner; No recent quit attempts | 14 item version of the PLS mean items score=7.75 (SD=.82) | nil | Photo of loved one (30 seconds) |
| Xu et al. (212b) | See Xu et al. (2011) | See Xu et al. (2011) | Still in relationship at 40 months post fMRI v no longer in relationship 40 months post fMRI | See Xu et al. (2011) |
| Scheele et al. (2013) | Study 1: n=20 (f=0); Mean age=25.1 (SD=3.3); Mean relationship duration=28.8 months (SD=15.4)  Study 2: n=20 (f=0); Mean age=26.6 (SD=3.8); Mean relationship duration=36.4 months (SD=25.3) | Study 1: PLS mean item score for experimental condition=6.33 (SD=0.96); Mean item score for placebo condition=6.46 (SD=1.21); MEIL Mean “Eros” score=7.19 (SD=0.99)  Study 2: PLS mean item score for experimental condition=6.46 (SD=0.1.36); Mean item score for placebo condition=6.49 (SD=1.27); MEIL Mean “Eros” score=6.98 (SD=0.99) | Nasal oxytocin administration (n=10) v placebo (n=10) | Photo of loved one |
| Song et al. (2015) | n=34; Mean age=21.23 (SD=2.45) | PLS (short version) mean=104.21 (SD=10.58); Mean duration of love=12.21 months (SD=3.33) | Ended-love group (n=34); Mean age=21.15 (SD=2.1); Duration since breakup (mean=10.41 months; SD=2.97); Length of relationship before breakup (mean=15.12 months; SD=9.91)  Single (never in love; n=32); Mean age=21.4 (SD=1.9) | nil |
| Wang et al. (2016) | n=22 (f=11); Mean age=22.51 (SD=2.19) | In love between 3 months and 3 years; Single item reporting passion in romantic love ranging from 1 to 10 mean 8.19 (SD=1.52) | nil | Photo of loved one-associated item (2-10 seconds) |
| Acevedo, Poulin, Collins, & Brown (2020) | T1: n=19 (f=11); Mean age=27.21 (SD=3.29); Mean relationship duration=4.11 years (SD=3.09); no children  T2 (mean time later=11.3): n=13 (f=7) | Eros subscale of LAS mean T1=6.33 (SD=0.32); Mean T2=6.17 (SD=0.55) | nil | Photo of loved one (20 seconds) |
| Wang et al. (2020) | n=34; Mean age=21.21 (SD=1.92) | PLS (short version) mean=104.21 (SD=10.58); Length of time in love=12.23 months (SD=2.95) | Singles (Never in love, not in a relationship); n=32 | nil |
| f=female; m=male; PLS=Passionate Love Scale; LAS=Love Attitudes Scale; MEIL=Marburg Attitude Scales towards Love Styles. **Notes.** Two fMRI studies (Yin et al., 2018; Yin et al., 2013) administered the PLS and administered passionate love-related stimuli but are not included in the table because the samples were not experiencing passionate love (PLS mean item score at least 5.73 as determined by the PLS creators, Hatfield & Sprecher 2011); Acevedo et al. (2012) study is included despite sample PLS mean item score less than 5.73 because long-term passionate love does not exhibit obsessive thinking and therefore scores are lower. | | | | |

**References**

Acevedo, B. P., Aron, A., Fisher, H. E., & Brown, L. L. (2012). Neural correlates of long-term intense romantic love. *Social Cognitive and Affective Neuroscience, 7*(2), 145-159. doi:10.1093/scan/nsq092

Acevedo, B. P., Poulin, M. J., Collins, N. L., & Brown, L. L. (2020). After the Honeymoon: Neural and Genetic Correlates of Romantic Love in Newlywed Marriages. *Frontiers in Psychology, 11*(634). doi:10.3389/fpsyg.2020.00634

Aron, A., Fisher, H., Mashek, D. J., Strong, G., Li, H. F., & Brown, L. L. (2005). Reward, motivation, and emotion systems associated with early-stage intense romantic love. *Journal of Neurophysiology, 94*(1), 327-337. doi:10.1152/jn.00838.2004

Bartels, A., & Zeki, S. (2000). The neural basis of romantic love. *Neuroreport, 11*(17), 3829-3834. doi:10.1097/00001756-200011270-00046

Bartels, A., & Zeki, S. (2004). The neural correlates of maternal and romantic love. *Neuroimage, 21*(3), 1155-1166. doi:10.1016/j.neuroimage.2003.11.003

Fisher, H. E., Brown, L. L., Aron, A., Strong, G., & Mashek, D. (2010). Reward, Addiction, and Emotion Regulation Systems Associated With Rejection in Love. *Journal of Neurophysiology, 104*(1), 51-60. doi:10.1152/jn.00784.2009

Hatfield, E., & Sprecher, S. (2011). The Passionate Love Scale. In T. D. Fisher, C. M. Davis, W. L. Yaber, & S. L. Davis (Eds.), *Handbook of sexuality-related measures: A compendium* (Third ed.). Thousand Oaks, CA: Taylor & Francis.

Kim, W., Kim, S., Jeong, J., Lee, K. U., Ahn, K. J., Chung, Y. A., . . . Chae, J. H. (2009). Temporal Changes in Functional Magnetic Resonance Imaging Activation of Heterosexual Couples for Visual Stimuli of Loved Partners. *Psychiatry Investigation, 6*(1), 19-25. doi:10.4306/pi.2009.6.1.19

Ortigue, S., Bianchi-Demicheli, F., Hamilton, A., & Grafton, S. T. (2007). The neural basis of love as a subliminal prime: An event-related functional magnetic resonance imaging study. *Journal of Cognitive Neuroscience, 19*(7), 1218-1230. doi:10.1162/jocn.2007.19.7.1218

Scheele, D., Wille, A., Kendrick, K. M., Stoffel-Wagner, B., Becker, B., Gunturkun, O., . . . Hurlemann, R. (2013). Oxytocin enhances brain reward system responses in men viewing the face of their female partner. *Proceedings of the National Academy of Sciences of the United States of America, 110*(50), 20308-20313. doi:10.1073/pnas.1314190110

Song, H. W., Zou, Z. L., Kou, J., Liu, Y., Yang, L. Z., Zilverstand, A., . . . Zhang, X. C. (2015). Love-related changes in the brain: a resting-state functional magnetic resonance imaging study. *Frontiers in Human Neuroscience, 9*, 13. doi:10.3389/fnhum.2015.00071

Stoessel, C., Stiller, J., Bleich, S., Boensch, D., Doerfler, A., Garcia, M., . . . Forster, C. (2011). Differences and Similarities on Neuronal Activities of People Being Happily and Unhappily in Love: A Functional Magnetic Resonance Imaging Study. *Neuropsychobiology, 64*(1), 52-60. doi:10.1159/000325076

Wang, C., Song, S. S., Uquillas, F. D., Zilverstand, A., Song, H. W., Chen, H., & Zou, Z. L. (2020). Altered brain network organization in romantic love as measured with resting-state fMRI and graph theory. *Brain Imaging and Behavior*. doi:10.1007/s11682-019-00226-0

Wang, Y., Zhang, Y. T., Chen, Y., Jing, F., Wang, Z. N., Hao, Y. R., . . . Zhang, X. C. (2016). Modulatory effect of romantic love on value estimation and its neural mechanism. *Neuroreport, 27*(5), 323-328. doi:10.1097/wnr.0000000000000541

Xu, X. M., Aron, A., Brown, L., Cao, G. K., Feng, T. Y., & Weng, X. C. (2011). Reward and Motivation Systems: A Brain Mapping Study of Early-Stage Intense Romantic Love in Chinese Participants. *Human Brain Mapping, 32*(2), 249-257. doi:10.1002/hbm.21017

Xu, X. M., Brown, L., Aron, A., Cao, G. K., Feng, T. Y., Acevedo, B., & Weng, X. C. (2012a). Regional brain activity during early-stage intense romantic love predicted relationship outcomes after 40 months: An fMRI assessment. *Neuroscience Letters, 526*(1), 33-38. doi:10.1016/j.neulet.2012.08.004

Xu, X. M., Wang, J., Aron, A., Lei, W., Westmaas, J. L., & Weng, X. C. (2012b). Intense Passionate Love Attenuates Cigarette Cue-Reactivity in Nicotine-Deprived Smokers: An fMRI Study. *Plos One, 7*(7), 9. doi:10.1371/journal.pone.0042235

Yin, J., Zhang, J. X., Xie, J., Zou, Z. L., & Huang, X. T. (2013). Gender Differences in Perception of Romance in Chinese College Students. *Plos One, 8*(10). doi:10.1371/journal.pone.0076294

Yin, J., Zou, Z. L., Song, H. W., Zhang, Z., Yang, B., & Huang, X. T. (2018). Cognition, emotion and reward networks associated with sex differences for romantic appraisals. *Scientific Reports, 8*, 11. doi:10.1038/s41598-018-21079-5

Younger, J., Aron, A., Parke, S., Chatterjee, N., & Mackey, S. (2010). Viewing Pictures of a Romantic Partner Reduces Experimental Pain: Involvement of Neural Reward Systems. *Plos One, 5*(10). doi:10.1371/journal.pone.0013309

Yin, J., Zou, Z. L., Song, H. W., Zhang, Z., Yang, B., & Huang, X. T. (2018). Cognition, emotion and reward networks associated with sex differences for romantic appraisals. *Scientific Reports, 8*, 11. doi:10.1038/s41598-018-21079-5

Younger, J., Aron, A., Parke, S., Chatterjee, N., & Mackey, S. (2010). Viewing Pictures of a Romantic Partner Reduces Experimental Pain: Involvement of Neural Reward Systems. *Plos One, 5*(10). doi:10.1371/journal.pone.0013309
