# Supplementary material for: Proximate and Ultimate Perspectives on Romantic Love
Source: Front Psychol. 2021 Apr 12;12:573123. doi: 10.3389/fpsyg.2021.573123 (PMC8074860; doi:10.3389/fpsyg.2021.573123)
Supplement: Supplementary file 3 [file Table_3.docx]

| **Table S3: Controlled endocrine studies investigating romantic love** | | | | | |
| --- | --- | --- | --- | --- | --- |
| **Study** | **Romantic love sample** | **Measure of romantic love** | **Control group(s)** | **Source** | **Factors measured** |
| Marazziti, Akiskal, Rossi, & Cassano (1999) | n=20 (17=f); Mean age=24 (SD=3); Medical students; Relationship commencing within past 6 months; No sexual intercourse with partner | At least 4 hours per day thinking about partner | n=20 (10=f); Mean age=29 (SD=2); No family or personal history of psychiatric disorder  n=20 (10=f); Mean age=29 (SD=6); Meet diagnostic criteria for DSM-IV OCD | Plasma; fasting; collected between 8-9am | Serotonin transporter |
| Marazziti & Canale (2004) | n=24 (12=f); Mean age=27 (SD=4); Relationship commencing within past 6 months; Mean relationship duration=3months (SD=1) | At least 4 hours per day thinking about partner; Specifically designed questionnaire | n=24 (12=f); Mean age=29 (SD=3); Long-lasting relationship or single; From same environment; Similar education levels | Serum; fasting; collected between 8-9am | Follicle-stimulating hormone, Luteinizing hormone, Estradiol; Progesterone; Testosterone; Dehydroepiandrosterone; Cortisol; Androstenedione |
| Emanuele et al. (2006) | n=58 (38=f); Mean age 24.2 (SD=3.8); Relationship commencing within past 6 months; No psychiatric disorder | At least 4 hours per day thinking about partner; semi-structured interview; PLS (short-version) scores > 85 (Mean=107.1; SD=14.9) | Long-lasting relationship group n=58 (38=f); Mean age=26.7 (SD=3.6); Mean relationship duration=49 months (SD=19 months)  No romantic relationship group n=58 (36=f); Mean age 26.8 (SD=3.7); | Plasma; post 14 hour fast; Female blood taken between day 3 and 5 of menses | Nerve growth factor; Brain-derived neurotrophic factor; Neurotrophin-3; Neurotrophin-4 |
| Dundon & Rellini (2012) | n=29 (f=29); Mean age approx. 19.76 (SD=1.51) | PLS; 3 items from screening at least a score of 17 | n=17; Mean age approx. 19.76 (SD=1.51) | Urine | Norepinephrine; Dopamine |
| Langeslag, van der Veen, & Fekkes (2012) | n=20 (f=10); Mean age f=19.7 (SD=2.3); Mean age m=21.3 (SD=2.7); In love for 9 months or less | General questionnaire (Intensity, obsessive thinking, love duration, relationship duration); PLS mean items score=6.8 (DS=1); PLS obsessive thinking items mean item score=5.8 (SD=1.6) | n=20 (f=10); Not in love | Plasma and Serum; Drawn between 8:45-10:30 am; after breakfast | Serotonin |
| Weisman, Schneiderman, Zagoory-Sharon, & Feldman (2015) | n=120 (f=60); Couples; “New Couples;” Mean relationship duration=23.93 weeks (SD=6.6 weeks); Under 36 years; n=79 provided sample | Passion subscale of STLS; Mean score=107 | n=35 (f-21); Single; n=34 provided sample | Saliva; immediately or within minutes of waking; During the day | Cortisol |
| Marazziti et al. (2017) | n=30 (f=15); Ages 20-40 years; Mean age 29.8 (SD=4); Romantic relationship less than 6 months (Mean=3 months; SD=1 month) | Specifically designed questionnaire | n=30 (f=15); Mean age=29.4 (SD=5); ages 20-40 years; Long-lasting relationship (more than 2 years; Mean=27months; SD=8 months) | Lymphocyte | Dopamine transporter |
| Sorokowski et al. (2019) | n=47 (f=47); ages 25-30 years; BMI < 30; No children | STLS | n=69 (f=69); Single; Not in love | Serum; 12h fast; between 7:30-9:30am; between day 2 and 4 of follicular phase | Estradiol; Luteinizing hormone; Follicle-stimulating hormone; Prolactin; Free testosterone; Cortisol |
| f=female; m=male; PLS=Passionate Love Scale; STLS= Sternberg’s Triangular Love Scale; BMI=body mass index | | | | | |

**References**

Dundon, C. M., & Rellini, A. H. (2012). Emotional States of Love Moderate the Association Between Catecholamines and Female Sexual Responses in the Laboratory. *Journal of Sexual Medicine, 9*(10), 2617-2630. doi:10.1111/j.1743-6109.2012.02799.x

Emanuele, E., Politi, P., Bianchi, M., Minoretti, P., Bertona, M., & Geroldi, D. (2006). Raised plasma nerve growth factor levels associated with early-stage romantic love. *Psychoneuroendocrinology, 31*(3), 288-294. doi:10.1016/j.psyneuen.2005.09.002

Langeslag, S. J. E., van der Veen, F. M., & Fekkes, D. (2012). Blood Levels of Serotonin Are Differentially Affected by Romantic Love in Men and Women. *Journal of Psychophysiology, 26*(2), 92-98. doi:10.1027/0269-8803/a000071

Marazziti, D., Akiskal, H. S., Rossi, A., & Cassano, G. B. (1999). Alteration of the platelet serotonin transporter in romantic love. *Psychological Medicine, 29*(3), 741-745. doi:10.1017/s0033291798007946

Marazziti, D., Baroni, S., Giannaccini, G., Piccinni, A., Mucci, F., Catena-Dell'Osso, M., . . . Dell'Osso, L. (2017). Decreased lymphocyte dopamine transporter in romantic lovers. *Cns Spectrums, 22*(3), 290-294. doi:10.1017/s109285291600050x

Marazziti, D., & Canale, D. (2004). Hormonal changes when falling in love. *Psychoneuroendocrinology, 29*(7), 931-936. doi:10.1016/j.psyneuen.2003.08.006

Sorokowski, P., Zelazniewicz, A., Nowak, J., Groyecka, A., Kaleta, M., Lech, W., . . . Pisanski, K. (2019). Romantic Love and Reproductive Hormones in Women. *International Journal of Environmental Research and Public Health, 16*(21). doi:10.3390/ijerph16214224

Weisman, O., Schneiderman, I., Zagoory-Sharon, O., & Feldman, R. (2015). Early Stage Romantic Love is Associated with Reduced Daily Cortisol Production. *Adaptive Human Behavior and Physiology, 1*(1), 41-53. doi:10.1007/s40750-014-0007-z
